# A 4-miRNA signature predicts the therapeutic outcome of glioblastoma

**DOI:** 10.18632/oncotarget.9945

**Published:** 2016-06-11

**Authors:** Maximilian Niyazi, Adriana Pitea, Michel Mittelbronn, Joachim Steinbach, Carsten Sticht, Franz Zehentmayr, Daniel Piehlmaier, Horst Zitzelsberger, Ute Ganswindt, Claus Rödel, Kirsten Lauber, Claus Belka, Kristian Unger

**Affiliations:** ^1^ Ludwig-Maximilians-University of Munich, Department of Radiation Oncology, Munich, Germany; ^2^ Research Unit of Radiation Cytogenetics, Helmholtz Zentrum München, Neuherberg, Germany; ^3^ Institute of Neurology (Edinger Institute), Goethe-University Frankfurt, Frankfurt/Main, Germany; ^4^ Dr. Senckenbergisches Institut für Neuroonkologie, Klinikum der J.W. Goethe-Universität, Frankfurt/Main, Germany; ^5^ Zentrum für Medizinische Forschung, Medizinische Fakultät Mannheim, Mannheim, Germany; ^6^ Department of Radiation Oncology, Paracelsus Medical University, Salzburg, Austria; ^7^ Department of Radiation Oncology, University Hospital, Frankfurt, Germany; ^8^ Clinical Cooperation Group Personalized Radiotherapy in Head and Neck Cancer, Helmholtz Zentrum München, Neuherberg, Germany; ^9^ German Cancer Consortium (DKTK), German Cancer Research Center (DKFZ), Heidelberg, Germany

**Keywords:** glioblastoma, miRNA, signature

## Abstract

Multimodal therapy of glioblastoma (GBM) reveals inter-individual variability in terms of treatment outcome. Here, we examined whether a miRNA signature can be defined for the *a priori* identification of patients with particularly poor prognosis.

FFPE sections from 36 GBM patients along with overall survival follow-up were collected retrospectively and subjected to miRNA signature identification from microarray data. A risk score based on the expression of the signature miRNAs and cox-proportional hazard coefficients was calculated for each patient followed by validation in a matched GBM subset of TCGA. Genes potentially regulated by the signature miRNAs were identified by a correlation approach followed by pathway analysis.

A prognostic 4-miRNA signature, independent of MGMT promoter methylation, age, and sex, was identified and a risk score was assigned to each patient that allowed defining two groups significantly differing in prognosis (p-value: 0.0001, median survival: 10.6 months and 15.1 months, hazard ratio = 3.8). The signature was technically validated by qRT-PCR and independently validated in an age- and sex-matched subset of standard-of-care treated patients of the TCGA GBM cohort (n=58). Pathway analysis suggested tumorigenesis-associated processes such as immune response, extracellular matrix organization, axon guidance, signalling by NGF, GPCR and Wnt. Here, we describe the identification and independent validation of a 4-miRNA signature that allows stratification of GBM patients into different prognostic groups in combination with one defined threshold and set of coefficients that could be utilized as diagnostic tool to identify GBM patients for improved and/or alternative treatment approaches.

## INTRODUCTION

Malignant gliomas account for approximately 70% of primary brain tumors diagnosed in adults. Median age at diagnosis is 64 years with men being more frequently affected than women [1].

Amongst all gliomas, glioblastoma (GBM) is the most common and aggressive form [2]. State-of-the-art treatment of GBM comprises surgical resection and adjuvant radiochemotherapy followed by maintenance chemotherapy. Implementation of temozolomide (TMZ) into the radiochemotherapeutic regime improved 2-year survival rates of patients with newly diagnosed malignant glioma (mainly GBM) from 11% to 27%, 3-year survival rates from 4% to 16%, and 5-year survival rates from 2% to 10% [3]. Unfortunately several phase III trials employing targeted agents such as bevacizumab (AVAglio & RTOG 0825) or cilengitide failed to show an improvement in overall survival [4, 5]. Thus, TMZ-based radiochemotherapy remains standard treatment for GBM. However, prognosis of most GBM patients still remains dismal with a high rate of local recurrence, emphasizing the clear need for further optimization [6]. At present, several strategies are being followed in this regard: Firstly, more elaborate imaging techniques as well as improved image-guidance during radiotherapy are being tested [7, 8]. Secondly, various molecularly designed substances are undergoing pre-clinical and clinical testing for their therapeutic efficacy in combination with radio(chemo)therapy [9, 10]. These targeted treatment approaches require molecular stratification of patients in order to identify the subgroups that can benefit most from a given strategy. Classical radiochemotherapy also displays wide inter-individual differences in terms of response and survival rates [11]. Accordingly, numerous efforts are undertaken in order to characterize the molecular mechanisms orchestrating therapy sensitivity and resistance and to identify prognostic and predictive markers.

So far, only few prognostic factors have been defined for GBM, including age and Eastern Cooperative Oncology Group (ECOG) score. In addition, involvement of the subventricular zone and extent of resection are known to be of prognostic importance [12]. More recently, the first molecular markers have been established. In this regard, methylation of the O6-methylguanine DNA-methyltransferase (MGMT) promoter region was recognized to be of positive predictive value for the efficacy of TMZ-based radiochemotherapy, and molecular profiling of long-term survivors disclosed the positive prognostic value of a proneural-like expression pattern linked to mutations in the genes encoding for iso-citrate dehydrogenases 1/2 (IDH1/2) [13].

During the last years, microRNAs (miRNAs) have increasingly received attention. With a high degree of promiscuity miRNAs target and regulate several mRNA species encoding for proteins involved in various signaling pathways [14]. Accumulating evidence indicates that miRNA expression signatures can serve as biomarkers for diagnosis and risk assessment of diverse malignancies, including GBM [15–20]. Given that the available prognostic markers can segregate GBM patients only to a limited extent, additional markers and/or signatures have to be defined. We focused on miRNA profiles, because the characterization of epigenetic alterations in the field of GBM research has hitherto been underrepresented and miRNA expression is well accessible from clinical routine diagnostic tissue specimen such as formalin-fixed paraffin-embedded (FFPE) tissue sections [21].

We sought to delineate a miRNA expression signature that is of predictive/prognostic value for overall survival in a retrospective cohort of 36 primary GBM patients who underwent adjuvant radiochemotherapy. Applying an iterative forward selection approach on miRNA microarray expression data, we identified a distinct signature comprising 4 miRNAs that was technically confirmed by quantitative real-time PCR (qRT-PCR) and independently validated in an age- and sex-matched data subset of a cohort of GBM patients who received standard-of-care treatment, obtained from The Cancer Genome Atlas (https://tcga-data.nci.nih.gov) project [22, 23, 3, 24]. Multivariate analysis revealed this signature to be independent of the MGMT promoter methylation status and of any other prognostic parameters that were available for our dataset.

## RESULTS

### Characterization of the patient cohort: survival data and univariate analysis

The MGMT promoter methylation status had no statistically significant influence on overall survival (p-value=0.763), although in Kaplan-Meier analysis a trend towards better survival could be observed in MGMT methylated patients. (Supplementary Figure S1) We also did not find statistically significant associations of overall survival with age (p-value=0.053) and sex (p-value=0.222).

### Extraction of a low complexity miRNA signature and evaluation of its prognostic significance for overall survival

**Figure 1:**
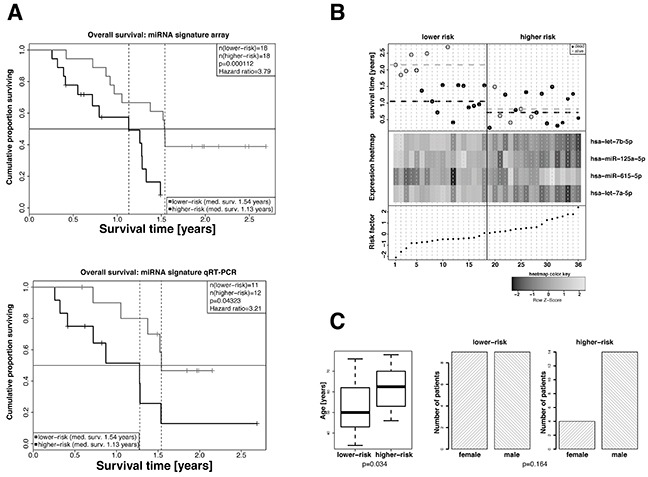
Extraction of a 4-miRNA signature as independent predictive marker for the overall survival of GBM patients in the exploratory cohort **A.** Kaplan-Meier overall survival analyses of high-risk and low-risk GBM patients. High-risk and low-risk patients were stratified based on the risk factors calculated from the cox-proportional hazard coefficients and the miRNA expression levels as measured in the microarray (left panel, 35 patients) or by qRT-PCR analyses (right panel, 19 patients). Hazard ratios and p-values were calculated by log-rank test. **B.** Overall survival (top panel), hierarchical cluster heat map of miRNA array expression levels (middle panel), and risk factors calculated on the basis of miRNA expression values and cox-proportional hazard coefficients (bottom panel) for all patients. miRNAs hsa-let-7a-5p, hsa-let-7b-5p and hsa-miR-125a-5p in patients of the higher-risk group show a tendency towards lower expression and that of hsa-miR-615-5p a tendency towards higher expression. The median risk factor value was used to classify high-risk and low-risk patients. **C.** Distribution of age (left panel) and sex (right panel) in high-risk and low-risk GBM patients. Statistical comparison was performed by Student's t-test or Fisher's exact test. The patients of the lower-risk group were statistically significantly older compared with that of the lower-risk group. The differences in the numbers of male and female patients of the lower- and higher-risk groups were not statistically significant.

We analyzed miRNA expression profiles in FFPE samples of our patient cohort and extracted a signature that consisted of the four miRNAs hsa-let-7a-5p, hsa-let-7b-5p, hsa-miR-125a-5p and hsa-miR-615-5p which was statistically significantly associated with overall survival (p-value=0.0048). The median risk score calculated from the expression levels of the signature miRNAs and the corresponding cox-proportional hazard coefficients (Table 1) separated the patients into a high- and a low-risk group. Cox regression analysis of the high- and the low-risk groups revealed a 3.79 fold increased risk of death (95% CI: 2.03-12.85) for the high-risk group compared to the low-risk group (p-value=0.000112). The median survival time was 13.5 months for patients of the high- risk group and 18.4 months for patients of the low-risk group, respectively. These results were visualized by Kaplan-Meier overall survival curves (Figure 1B). Univariate testing of the individual miRNAs within the signature revealed p-values in the range between 0.0015 and 0.016, indicating that each single miRNA was able to statistically significantly predict overall survival. Expressions of miRNAs hsa-let-7a-5p, hsa-let-7b-5p and hsa-miR-125a-5p positively correlated with overall survival and and that of hsa-miR-615-5p negatively correlated with overall survival. Figure 1A summarizes the survival data of the patients in relation to the calculated risk scores and expression levels. When including MGMT promoter methylation status in a multivariate cox-proportional hazard model, its contribution to the model was not statistically significant, thereby suggesting that the identified miRNA signature performs independently of the MGMT promoter methylation status. Moreover, the other available clinical parameters, such as sex and age were not associated with the calculated risk-score and also did not statistically significantly contribute to the multivariate model when included. A detailed representation of the results can be found in Table 1. Further, patients in the high-risk group were older compared to that in the low-risk group. Concerning distribution of sex there were no differences between the high- and the low-risk groups (Figure 1C).

**Table 1:** Results of multivariate cox-proportional hazard analysis of 4-miRNA risk score, age, sex and MGMT promoter methylation status

Cohort	Model	Hazard ratios of parameters	Confidence intervals of hazard ratios	p-values of contributions of parameters to model	p-value of model
Discovery	4-miRNA risk-score	3.8	1.47-9.75	0.00574	0.00434
	MGMTmeth	0.9	0.39-2	0.7637	0.76298
	4-miRNA risk-score+MGMTmeth	3.8,0.9	1.48-9.81/0.38-1.93	0.00558,0.70124	0.0159
	Sex	1.7	0.72-3.86	0.22874	0.22151
	4-miRNA risk-score+Sex	3.6,1.3	1.36-9.32/0.57-3.16	0.00982,0.50574	0.01367
	Age	1	1-1.07	0.05469	0.05267
	4-miRNA risk-score+Age	3.5,1	1.35-9.12/0.99-1.07	0.00979,0.10002	0.00439
	4-miRNA risk-score+MGMTmeth+Sex+Age	3.3,0.9,1,1.4	1.23-8.62/0.38-2.15/0.99-1.07/0.59-3.44	0.01765,0.82414,0.11408,0.43099	0.02158
Validation	4-miRNA risk-score	2.4	1.03-5.69	0.04207	0.04247
	MGMTmeth_1	0.5	0.18-1.27	0.13798	0.12236
	MGMTmeth_2	0.5	0.21-1.32	0.16924	0.15812
	4-miRNA risk-score+MGMTmeth_1	3.1,0.4	1.24-7.73/0.12-1.01	0.0156,0.05204	0.01532
	4-miRNA risk-score+MGMTmeth_2	2.2,0.6	0.93-5.28/0.24-1.55	0.07096,0.29498	0.07214
	Sex	1.5	0.57-3.8	0.41996	0.41121
	4-miRNA risk-score+Sex	1	1.36-0.9-9.31-7.64	0.00947,0.07811	0.02402
	Age	1	0.94-1.04	0.63117	0.63333
	4-miRNA risk-score+Age	0	1.09-0.93-6.25-1.03	0.03098,0.37596	0.08763
	4-miRNA risk-score+MGMTmeth_1+Sex+Age	4.1,0.5,2.1,1	1.44-11.49/0.16-1.79	0.008,0.30791, 0.24802,0.37623	0.03941
	4-miRNA risk-score+MGMTmeth_2+Sex+Age	4.5,1,3,1	1.41-14.67/0.34-3.09	0.01139,0.95454,0.08428,0.22869	0.06184

### Independent in silico validation of the detected miRNA signature

**Figure 2:**
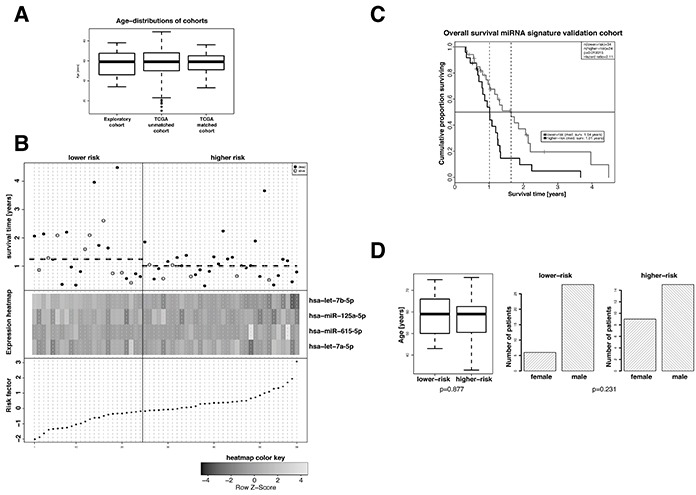
Evaluation of the prognostic value of the extracted 4-miRNA signature in an age- and sex-matched subgroup of standard-of-care treated patients of the TCGA GBM dataset **A.** Age distribution in the exploratory cohort and the TCGA GBM cohort before and after age matching. **B.** Overall survival (top panel), hierarchical cluster heat map of miRNA expression levels (middle panel), and risk factors for patients of the age- and sex-matched TCGA GBM cohort. The median risk factor value was used to classify high-risk and low-risk patients. miRNAs hsa-let-7a-5p, hsa-let-7b-5p and hsa-miR-125a-5p in patients of the higher-risk group show a slight tendency towards lower expression and that of hsa-miR-615-5p a slight tendency towards higher expression. C. Kaplan-Meier overall survival analyses of high-risk and low-risk standard-of-care treated patients of the age- and sex-matched TCGA GBM cohort. Classification of high-risk and low-risk patients was performed on the basis of the risk factors calculated from the cox-proportional hazard coefficients (Table 2) and the miRNA expression levels. Hazard ratios and p-values were calculated by log-rank test. D. Distribution of age (left panel) and sex (right panel) in high-risk and low-risk patients of the age- and sex-matched TCGA GBM cohort. Student's t-test and Fisher's exact test were employed for statistical comparison as depicted.

For the purpose of independent validation the miRNA signature was tested in an age-matched miRNA data subset of standard-of-care treated patients (see Supplementary Table S1) of an independent GBM cohort downloaded from the TCGA database [25]. The high- and the low-risk groups were defined by using the median risk score of the discovery set (0.07811832) to dichotomize the patients of the validation set. The resulting cox-proportional hazard model revealed a hazard ratio of 2.11 (95% CI 1.13-3.91) and a p-value of 0.02 (Figure 2C). Figure 2B summarizes the survival data of the patients of the validation cohort in relation to calculated risk scores and expression levels. Also for the validation cohort no statistically significant association was found between high- and low-risk groups and the parameters age and sex (Figure 2D). Univariate testing of the MGMT promoter methylation status derived from two DNA methylation array probes that have been shown previously to reliably measure MGMT promoter methylation status for association with overall survival was conducted. No statistically significant association was observed (cg12434587: p.value: 0.122/hazard-ratio: 0.48, cg12981137: p-value: 0.16/hazard-ratio: 0.48) although in Kaplan-Meier analysis a trend towards better survival in MGMT promoter methylation positive was also observable here (Supplementary Figure S2). No differences in the distribution of age and sex were observed in the high- and low-risk groups identified in the validation cohort. Also, including in the multivariate cox model MGMT promoter methylation status did not show statistical significant influence on survival (Table 1).

### Technical validation of signature by qRT-PCR

In order to technically validate the 4-miRNA signature and to support potential applicability in clinical routine diagnostics, we measured the expression of the four miRNAs in a subset of samples (n=23), for which residual material was available by qRT-PCR. Analogous cox-proportional hazard analysis with the qRT-PCR data confirmed the results obtained with the miRNA array data. Patients of the high-risk group revealed significantly impaired overall survival (p-value=0.043) and a hazard-ratio of 3.21 (95% CI 1.02-10.16) as compared to patients of the low-risk group. (Figure 1A).

### miRNA-mRNA correlation and pathway enrichment analysis

**Figure 3:**
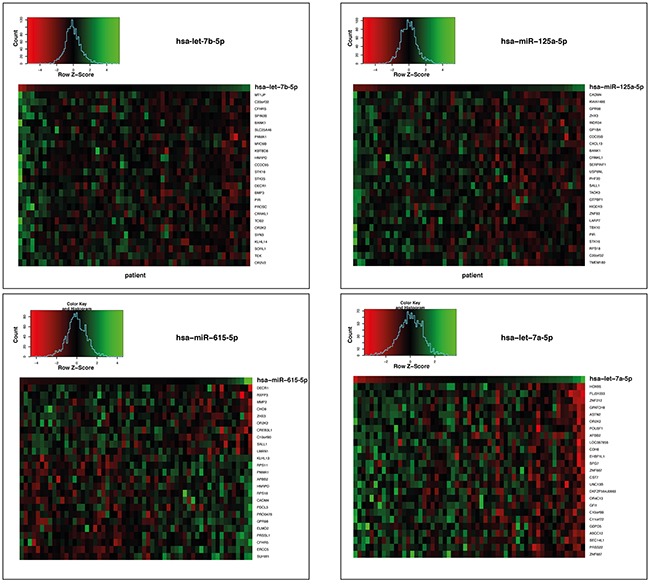
Heatmaps of the gene expressions correlating with the 4 miRNAs hsa-let-7b-5p, hsa-miR-125a-5p, hsa-miR-615-5p and hsa-let-7a-5p the age- and sex-matched TCGA GBM cohort of standard-of-care treated patients Genes whose expression levels statistically significantly correlated (p < 0.01) with the respective miRNA expression levels are shown. Every column represents an individual patient. Data are ordered from left to right by increasing miRNA expression.

For hsa-let-7b-5p we identified 104 significantly correlating genes (53 negative and 51 positive correlations), for hsa-miR-125a-5p 112 genes (35 negative and 77 positive correlations), for hsa-miR-615-5p 26 genes (10 negative and 16 positive correlations) and for hsa-let-7a-5p 412 genes (245 negative and 167 positive correlations). The overlap between genes correlating with expression of the signature miRNAs was sparse (Supplementary Figure S1). Heatmaps of the top 25 miRNA-mRNA correlations with regard to absolute correlation coefficients are depicted in Figure 3. Interestingly, whereas hsa-let-7b-5p, hsa-miR-125a-5p, and hsa-let-7a-5p displayed predominantly negative correlations among the top correlations as to be expected, hsa-miR-615-5p also showed positive correlations. All genes with significant correlations (Pearson correlation test, p-value < 0.01) were combined into one list of genes (n=654; Supplementary Table S2) and subjected to pathway enrichment analysis. In total, 28 statistically significant pathways were identified (Supplementary Table S3), and the top ten of these (i.e. Transmembrane transport of small molecules, Innate Immune System, Extracellular matrix organization, Axon guidance, Signalling by NGF, Developmental Biology, Neuronal System, GPCR downstream signaling, Signaling by GPCR and Signaling by Wnt) were considered for interpretation of results.

## DISCUSSION

GBM patients, who receive surgical resection and postoperative radio(chemo)therapy, reveal profound differences in terms of overall survival which motivated us to search for a miRNA signature that allows the identification of patients with specifically poor prognosis independently of any other outcome-associated parameters. Moreover, we intended to investigate such a signature with regard to the molecular mechanisms potentially underlying the poor outcome of GBM patients.

To this end, we performed miRNA microarray analysis followed by low-complexity miRNA signature identification. We could extract a 4-miRNA signature which, with high statistical significance, allowed differentiating between high- and low-risk GBM patients independently of the MGMT methylation status. Technical validation by qRT-PCR confirmed the microarray data results. Most importantly, the prognostic value of the signature could be confirmed by independent validation in a large subset of the TCGA study on GBM [25].

At present, the role of individual miRNAs in GBM is poorly understood. miRNAs are small non-coding regulatory RNAs that reduce stability and/or inhibit translation of target mRNAs with full or partial sequence-complementarity [14]. In this sense, they are important post-transcriptional regulators and play essential roles in the pathogenesis, development, and progression of cancer as well as in the response to therapy [26–28].

It has been shown that GBMs display distinct miRNA expression signatures, and several studies have linked these miRNA alterations to hallmarks of GBM, including proliferation, survival, invasion, angiogenesis, and stem cell-like behavior [29]. Moreover, resistance to TMZ might be associated with miRNA deregulation [30]. In this regard, Ciafre et al. studied the expression of 245 microRNAs in GBM in comparison to normal brain tissue using a microarray technique [31] in comparison to normal brain tissue. This approach enabled the identification of miRNAs whose expression levels were significantly altered in tumor tissue compared to peripheral brain tissue of the same patient, including miR-221, which was strongly up-regulated in GBM, and a set of brain-enriched miRNAs (miR-128, miR-181a, miR-181b, and miR-181c), which were down-regulated in GBM [32]. Very recently, a number of prognostic miRNA signatures have been reported for GBM [33–35, 15, 36, 37, 18–20, 38, 39]. We compared these signatures with our signature in terms of complexity, independent validation, and the approach used for identification of the signature. One important feature of molecular signatures is their level of complexity (i.e. the number of miRNAs), which should be most optimal with regard to prediction performance but at the same time should not overfit the data. For data sets with moderate dimensionality such as miRNA microarray data sets with typically a few hundreds of miRNAs expressed, the number of features contained in a signature should be of low complexity and in the range of smaller than 10. From the above cited studies, only five extracted a miRNA signature with low complexity that have been subsequently validated in an independent cohort [15, 37, 18–20]. Cheng et al. [15] focused on MGMT promoter methylation positive tumors only and defined a 5-miRNA signature that was validated in an appropriate subset of the so-called Chinese Glioma Genome Atlas. In contrast, our signature was developed using data from both MGMT promoter methylation-positive and -negative tumors. The signatures described by Li et al. were developed for each of the five molecular GBM subtypes as defined by transcriptomic profiling of TCGA GBM cases [37, 40]. Only the signature for the ‘mesenchymal’ subtype consisting of five miRNAs was independently validated in a set of GBM tissues [37]. Our signature, in contrast, is not limited to this molecular subtype only. The small-noncoding RNAs described in Manterola et al. [18] allow molecular diagnosis of GBM using blood serum samples but the authors did not show usability with regard to outcome prediction whilst our signature was particularly developed for the purpose of predicting survival outcome. In a study by Shou et al. [20] three miRNAs were presented that statistically significantly allow differentiation into groups of patients with favorable and unfavorable prognosis. However, this approach was limited to separate and univariate analysis for each of the three miRNAs and the study did not include an independent validation of the results. The report by Sana et al. is most comparable to our study and introduced a 6-miRNA-signature which was also validated in the TCGA GBM data set [19]. However, contrary to our study Sana et al. used different thresholds for the calculated risk scores of the discovery and the validation set. Most importantly, the thresholds were chosen in such a way that they statistically significantly separated the two resulting groups of patients [19]. This can be interpreted as a technical drawback, and the approach has to be considered as a biased one. In strong contrast, we applied the same cox-proportional hazard coefficients and the same risk score thresholds to all of our three datasets (discovery Febit microarray, discovery qRT-PCR and validation Agilent microarray). This renders our 4-miRNA-signature, in principle, applicable to any other data set regardless of the platform the data were generated with.

Comparing our signature with that of the above mentioned studies did show no overlapping miRNAs of our signature with of the published ones. This can be explained by the fact that signature identification is very much dependent on the methodology used, the dataset with regard to the specific selection criteria of patients and the platform that is used for measurements. Since all mentioned studies vary with regard to these parameters one could not expect overlap of our signature with the published ones or overlap between the published ones.

Besides its prognostic value, it is of major interest to understand the impact of the 4-miRNA-signature on the biological characteristics of GBM. Our panel of miRNAs consists of hsa-let-7a-5p, hsa-let-7b-5p, hsa-miR-125a-5p and hsa-miR-615-5p. Two miRNAs of the signature belong to the let-7 family, which is very well known for its tumor suppressor function in various cancer entities [41]. The two let-7 miRNAs hsa-let-7a-5p and hsa-let-7b-5p showed a tendency towards higher expression levels in the low-risk compared to the high-risk group of patients, which is in line with the concept of their involvement in tumor suppression. miR-125a-5p was described as a tumor suppressor in GBM only recently. It is engaged in the repression of target genes of the TAZ (transcriptional co-activator with PDZ-binding motif) transcription factor, including connective tissue growth factor (CTGF) and survivin [42]. Our analysis revealed a tendency towards higher miR-125a-5p expression levels in the low-risk compared to the high-risk group of patients, again supporting its role in tumor suppression. hsa-miR-615-5p was described to act as tumor suppressor in pancreatic ductal adenocarcinoma [17]. In our analyses, a clear tendency towards higher or lower expression levels of hsa-miR-615-5p in the low- and high-risk group of patients was not observable. Therefore, conclusions concerning its tumorsuppressive role in GBM cannot be drawn. Overall, our 4-miRNA-signature reflects trends of higher expression levels of tumor suppressive miRNAs in low-risk GBMs, supporting the notion that these GBMs exhibit a lower degree of malignancy due to operational tumor suppressive mechanisms. In order to gain insights into the putative functional role of the four signature miRNAs, we conducted miRNA-transcriptome correlation analyses and obtained 654 genes, whose expression levels were positively or negatively correlated with that of the miRNAs. We deliberately followed this approach to identify direct and indirect regulatory effects of the signature miRNAs on the transcriptome. An alternative approach would have been to utilize miRNA target prediction. This, however, relies strongly on the prediction algorithm and the database that are used, and databases providing information on in vitro validated miRNA targets are still limited with regard to the number of miRNAs they provide information on [43]. The genes that were identified in our correlation approach were subjected to pathway enrichment analyses, which disclosed the top ten pathways Transmembrane transport of small molecules, Innate Immune System, Extracellular matrix organization, Axon guidance, Signalling by NGF, Developmental Biology, Neuronal System, GPCR downstream signaling, Signaling by GPCR and Signaling by Wnt all of which very well known in the context of glioblastoma tumorigenesis. These results suggest that our 4-miRNA signature regulates genes that are well known to be involved in the tumorigenesis, progression and migration of GBM and may potentially act as druggable targets in an alternative treatment approach.

## CONCLUSIONS

In the present study, we extracted and validated a 4-miRNA-signature, which allows to differentiate GBM patients undergoing surgical resection and subsequent radio(chemo)therapy with favorable and poor prognosis. This signature may serve as a potential new marker for patient stratification independent of the MGMT methylation status. It may furthermore pave the way for personalized treatment approaches based on measurements that are well feasible in GBM biopsies in the clinical routine. Patients with a high-risk score are likely not profiting from standard-of-care treatment and therefore the 4-miRNA signature could be used to identify patients who require therapy intensification. Compared to existing GBM miRNA signatures the herein presented signature is of lower complexity, was independently validated, and appears to be in principle applicable to any data set containing expression values of the four signature miRNAs regardless of the platform they were generated with.

## MATERIALS AND METHODS

For a detailed description of Material and Methods sections ‘Patient characteristics’, ‘miRNA array analysis’, ‘Technical validation of the 4-miRNA signature by qRT-PCR’ and ‘miRNA-mRNA correlation and gene set enrichment analysis’ see Supplementary Data.

### Patient characteristics

We examined FFPE tissue samples of a non-selected, retrospective cohort of patients who were consecutively treated at the University hospital Frankfurt between 1/2009 and 12/2010. Ethics approval (4/09) was given by the ethics committee of the medical faculty of the Johann-Goethe University (Frankfurt am Main, Germany). Only patients who underwent surgical resection and post-operative radio(chemo)therapy were included into the analyses. Patients underwent resection and adjuvant radiotherapy, regularly combined with TMZ according to the EORTC/NCIC26981/22981-NCIC CE3 protocol if no contraindications were present (for details see Table 2) [3, 24]. The median overall survival time of this patient cohort was 1.28 years with a median follow-up of 1.99 years (95%-CI, 634 - 816 days). MGMT promoter methylation status was available for all 36 cases (see Table 1). Karnofsky performance status (KPS) score and associated recursive partitioning analysis (RPA) class had not been collected systematically, and no data on the extent of resection was available. For independent validation, the miRNA expression dataset from an age- and sex-matched subset (n=58) of the TCGA GBM cohort (n=357) was used. The subset resulted after adjusting the distribution of age of the whole TCGA GBM dataset to that of our discovery cohort (Table 3) and only selecting patients that were treated according to standard-of-care.

**Table 2:** Cox-proportional hazard coefficients used in risk score calculation

miRNA	coefficient
hsa-let-7b-5p	−0.9669152
hsa-miR-125a-5p	−0.2821517
hsa-miR-615-5p	0.3254795
hsa-let-7a-5p	0.5059587

**Table 3:** Clinical characteristics of discovery cohort

Characteristic	Patients (N=36)
Sex	
Male	23 (63.9 %)
Female	13 (36.1 %)
Median Age [y]	59 (34-78)
Age Category	
< 50 y	13 (36.1 %)
≥ 50 y	23 (63.9 %)
MGMT promoter methylation status	
Methylated	18 (50.0 %)
Unmethylated	18 (50.0 %)
Secondary Malignisation	
Yes	12 (33.3 %)
No	24 (66.7 %)
Concomitant Temozolomide	
Yes	32 (88.9 %)
No	1 (2.8 %)
Unknown	3 (8.3 %)
Median adjuvant TMZ cycles	6 (0−20)

### miRNA array analysis

miRNA analysis was carried out using the Geniom Biochip MPEA homo sapiens biochips containing 1223 miRNA probes (CBC, Heidelberg, Germany). FFPE sample preparation, hybridization, washing and scanning of arrays was performed as described previously [44]. We applied ‘winsorized mean’ scaling on normalized data with exclusion of 30% of the top and bottom values.

### TCGA glioblastoma miRNA data set

The validation data set was constructed from miRNA microarray profiles of the matched subset of patients from the TCGA GBM cohort. Data were generated by the University of North Carolina Cancer Genomic Characterization Center (CGCC) using the Agilent 8×15K Human miRNA-specific microarray platform [22]. For the analysis level 3 data were used and in order to allow comparability of the data set with the discovery data set scaling with ‘winsorized mean’ was applied as described above.

### miRNA signature robust selection

In order to search for a miRNA signature in miRNA expression data set of the discovery cohort associated with patient survival, the R package rbsurv was used [45]. The forward-selection algorithm implemented in the package computed the partial likelihood of the Cox model for a sequential selection of miRNAs. The best performing model was chosen based on the Akaike Information Criterion (AIC), which allowed to determine the best trade-off between the complexity of a model and its goodness of fit.

### Calculation of risk scores

The Cox model coefficients (Table 2) were multiplied with the scaled expression values of appropriate miRNAs and the products were summed up resulting in an individual risk score for each patient. The median risk score of all patients (0.07811832) was used as a cut-off for defining a high-risk (> median risk score) and a low-risk group (< median risk score). Subsequently, the log-rank test was used to test whether the differences in overall survival times between the resulting two groups were statistically significant (p-value threshold: 0.05). Further, Kaplan-Meier survival curves were plotted for the two groups and the hazard ratio was calculated. The influence of the available known risk factors age, sex, and MGMT promoter methylation status was assessed univariately and by inclusion into the multivariate cox-proportional hazard model.

### Independent *in silico* validation of the 4-miRNA signature

For each of the 58 included TCGA GBM patients (Supplementary Table S1) we calculated a risk score by building the sum of the products of the expressions of the four miRNAs of the signature and the coxproportional hazard coefficients obtained from the initial dataset for each of the miRNAs (Table 2).

The patients were assigned to high- and low-risk groups by using the same threshold (0.07811832) that was defined for the discovery data set. The resulting two groups were tested for differential survival outcome using log-rank test.

### Technical validation of the 4-miRNA signature by qRT-PCR

MiScript primer assays (QIAGEN, MD, USA) for the four miRNAs of the signature were used for relative quantification along with a reference assay for the small RNA SNORD61. The relative expression values in combination with the cox-proportional hazard coefficients were used to calculate a risk score for every patient. The patients were dichotomized into a low- and high-risk group using the risk score threshold from the discovery cohort (0.07811832) and the resulting two groups were tested for differences in overall survival using log-rank test of the resulting cox-proportinal hazard model.

### miRNA-mRNA correlation and gene set enrichment analysis

In order to investigate the impact of the four signature miRNAs on the transcriptome level we downloaded the transcriptome data (level 3) of the cases matching the miRNAs data set (n=132) from the TCGA database and calculated correlations between the four signature miRNAs and expression levels of all genes. Genes that statistically significantly correlated with the signature miRNAs were subjected to pathway enrichment analysis.

### MGMT promoter methylation typing

For the discovery cohort determination of MGMT promoter methylation was performed using both methylation-specific PCR and sequencing analysis as described previously [46, 47].

For the TCGA validation cohort no systematic assessment of the MGMT promoter methylation status was available. However, in order to determine methylation of the MGMT promoter we followed an approach published by Bady et al. from methylation array data [48]. The resulting MGMT promoter-positive and -negative groups were then subsequently tested for association with survival univariately and multivariately.

## SUPPLEMENTARY FIGURE AND TABLES








