# Supplementary material for: A 4-miRNA signature predicts the therapeutic outcome of glioblastoma
Source: Oncotarget. 2016 Jun 11;7(29):45764–75. doi: 10.18632/oncotarget.9945 (PMC5216759; doi:10.18632/oncotarget.9945)
Supplement: Supplementary file 1 [file oncotarget-07-45764-s001.pdf]

A 4-miRNA signature predicts the therapeutic outcome of glioblastoma

SUPPLEMENTARY FIGURE AND TABLES

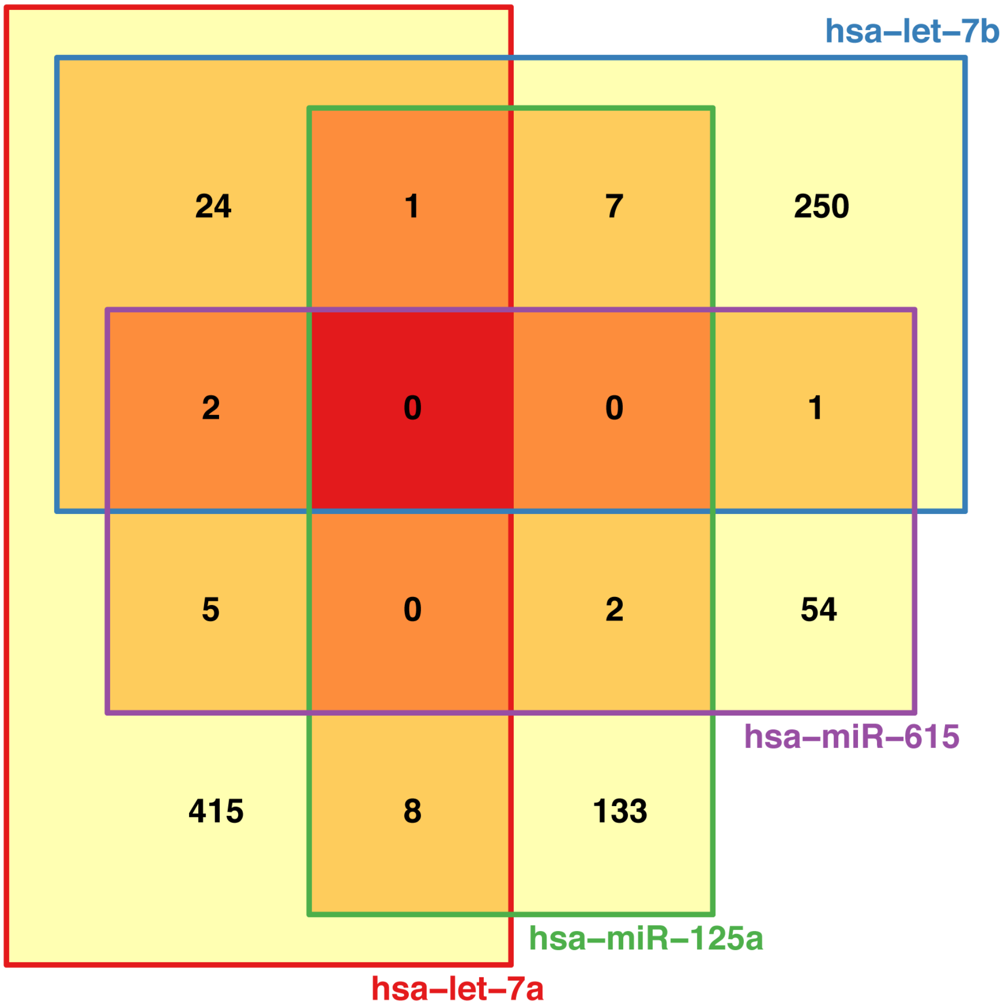

Supplementary Figure S1: Venn diagram showing overlaps between genes correlating with expression of the four signature miRNAs hsa-let-7a-5p, hsa-let-7b-5p, hsa-miR-615-5p and hsa-miR-125a-5p. Only sparse overlap between lists of genes were detectable despite genes regulated by hsa-let-7a-5p and hsa-let-7b-5p which showed 24 commonly regulated genes.

Supplementary Table S1: Clinical data of TCGA GBM validation subset.

See Supplementary File 1

Supplementary Table S2: Results of correlation analysis of signature miRNAs with mRNA expression in TCGA GBM validation subset.

See Supplementary File 2

Supplementary Table S3: Pathway enrichment analysis with genes significantly correlating with signature miRNAs.

See Supplementary File 3
