# Supplementary material for: A 4-miRNA signature predicts the therapeutic outcome of glioblastoma
Source: Oncotarget. 2016 Jun 11;7(29):45764–75. doi: 10.18632/oncotarget.9945 (PMC5216759; doi:10.18632/oncotarget.9945)
Supplement: Supplementary file 2 [file oncotarget-07-45764-s002.docx]

Part 1

| CASE_ID | BARCODE | AGE | DAYS_TO_BIRTH | DAYS_TO_DEATH | DAYS_TO_INITIAL_PATHOLOGIC_DIAGNOSIS | DFS_MONTHS | DFS_STATUS | ECOG_SCORE |
| --- | --- | --- | --- | --- | --- | --- | --- | --- |
| TCGA-32-5222-01 | TCGA-32-5222 | 66 | -24134 | NA | 0 | 6,34 | Recurred/Progressed | NA |
| TCGA-27-1831-01 | TCGA-27-1831 | 66 | -24447 | 505 | 0 | 4,73 | Recurred/Progressed | 1 |
| TCGA-08-0531-01 | TCGA-08-0531 | 64 | -23482 | 230 | 0 | NA | NA | NA |
| TCGA-06-0133-01 | TCGA-06-0133 | 64 | -23402 | 435 | 0 | 2,56 | Recurred/Progressed | NA |
| TCGA-28-2506-01 | TCGA-28-2506 | 63 | -23292 | NA | 0 | 2,76 | Recurred/Progressed | NA |
| TCGA-28-1751-01 | TCGA-28-1751 | 61 | -22291 | NA | 0 | 7,19 | Recurred/Progressed | 1 |
| TCGA-06-0125-01 | TCGA-06-0125 | 63 | -23343 | 1448 | 0 | 26,18 | Recurred/Progressed | NA |
| TCGA-12-3648-01 | TCGA-12-3648 | 61 | -22463 | 819 | 0 | 2,83 | Recurred/Progressed | NA |
| TCGA-06-6389-01 | TCGA-06-6389 | 49 | -18248 | NA | 0 | 7,79 | DiseaseFree | NA |
| TCGA-12-1599-01 | TCGA-12-1599 | 47 | -17432 | 781 | 0 | 2,92 | Recurred/Progressed | NA |
| TCGA-08-0349-01 | TCGA-08-0349 | 46 | -16964 | 298 | 0 | 3,06 | Recurred/Progressed | NA |
| TCGA-12-0772-01 | TCGA-12-0772 | 46 | -17019 | 1638 | 0 | 19,94 | Recurred/Progressed | NA |
| TCGA-12-3650-01 | TCGA-12-3650 | 46 | -17138 | 333 | 0 | 7,85 | Recurred/Progressed | NA |
| TCGA-12-0778-01 | TCGA-12-0778 | 53 | -19460 | 454 | 0 | 7,62 | Recurred/Progressed | NA |
| TCGA-08-0525-01 | TCGA-08-0525 | 52 | -19013 | 486 | 0 | 11,27 | Recurred/Progressed | NA |
| TCGA-02-0106-01 | TCGA-02-0106 | 54 | -19883 | 355 | 0 | 6,44 | Recurred/Progressed | NA |
| TCGA-02-0115-01 | TCGA-02-0115 | 52 | -19257 | 476 | 0 | 2,99 | Recurred/Progressed | NA |
| TCGA-12-0776-01 | TCGA-12-0776 | 52 | -19228 | 296 | 0 | 4,17 | Recurred/Progressed | NA |
| TCGA-27-2518-01 | TCGA-27-2518 | 52 | -19130 | 753 | 0 | 17,71 | Recurred/Progressed | 1 |
| TCGA-12-0769-01 | TCGA-12-0769 | 52 | -19171 | 458 | 0 | 12,42 | Recurred/Progressed | NA |
| TCGA-02-2485-01 | TCGA-02-2485 | 53 | -19494 | NA | 0 | 6,11 | Recurred/Progressed | NA |
| TCGA-08-0510-01 | TCGA-08-0510 | 75 | -27675 | 130 | 0 | 2,96 | Recurred/Progressed | NA |
| TCGA-06-1087-01 | TCGA-06-1087 | 75 | -27658 | 123 | 0 | 3,58 | Recurred/Progressed | NA |
| TCGA-41-2575-01 | TCGA-41-2575 | 75 | -27587 | 290 | 0 | 4,3 | Recurred/Progressed | NA |
| TCGA-27-2519-01 | TCGA-27-2519 | 48 | -17715 | NA | 0 | 8,41 | Recurred/Progressed | 1 |
| TCGA-02-0064-01 | TCGA-02-0064 | 50 | -18280 | 600 | 0 | 16,29 | Recurred/Progressed | NA |
| TCGA-12-0618-01 | TCGA-12-0618 | 49 | -18071 | 395 | 0 | NA | NA | NA |
| TCGA-08-0358-01 | TCGA-08-0358 | 50 | -18383 | 678 | 0 | 8,67 | Recurred/Progressed | NA |
| TCGA-08-0244-01 | TCGA-08-0244 | 62 | -22684 | 690 | 0 | 15,9 | Recurred/Progressed | NA |
| TCGA-32-2491-01 | TCGA-32-2491 | 63 | -23131 | 372 | 0 | 2,3 | Recurred/Progressed | NA |
| TCGA-02-0075-01 | TCGA-02-0075 | 63 | -23205 | 634 | 0 | 11,04 | Recurred/Progressed | NA |
| TCGA-27-2528-01 | TCGA-27-2528 | 62 | -22867 | 480 | 0 | 2,37 | Recurred/Progressed | 1 |
| TCGA-08-0348-01 | TCGA-08-0348 | 63 | -23373 | 370 | 0 | 6,5 | Recurred/Progressed | NA |
| TCGA-02-0027-01 | TCGA-02-0027 | 33 | -12369 | 370 | 0 | 8,44 | Recurred/Progressed | NA |
| TCGA-26-1442-01 | TCGA-26-1442 | 43 | -15950 | NA | 0 | 31,31 | DiseaseFree | 1 |
| TCGA-08-0390-01 | TCGA-08-0390 | 69 | -25304 | 425 | 0 | NA | NA | NA |
| TCGA-02-0070-01 | TCGA-02-0070 | 70 | -25916 | NA | 0 | 25,03 | Recurred/Progressed | NA |
| TCGA-12-0775-01 | TCGA-12-0775 | 75 | -27565 | 232 | 0 | 3,09 | Recurred/Progressed | NA |
| TCGA-19-5951-01 | TCGA-19-5951 | 76 | -27995 | 244 | 0 | NA | NA | NA |
| TCGA-12-3646-01 | TCGA-12-3646 | 59 | -21855 | 1339 | 0 | 43,2 | Recurred/Progressed | NA |
| TCGA-12-5295-01 | TCGA-12-5295 | 60 | -22161 | 454 | 0 | 13,11 | Recurred/Progressed | 1 |
| TCGA-02-0086-01 | TCGA-02-0086 | 45 | -16763 | 268 | 0 | 3,19 | Recurred/Progressed | NA |
| TCGA-06-5858-01 | TCGA-06-5858 | 45 | -16662 | NA | 0 | 3,19 | Recurred/Progressed | 0 |
| TCGA-06-0644-01 | TCGA-06-0644 | 71 | -26247 | NA | 0 | 2,79 | Recurred/Progressed | NA |
| TCGA-06-5414-01 | TCGA-06-5414 | 61 | -22527 | NA | 0 | 5,49 | Recurred/Progressed | NA |
| TCGA-06-6701-01 | TCGA-06-6701 | 60 | -22051 | NA | 0 | 4,96 | DiseaseFree | NA |
| TCGA-12-0620-01 | TCGA-12-0620 | 57 | -21069 | 318 | 0 | NA | NA | NA |
| TCGA-06-6390-01 | TCGA-06-6390 | 58 | -21328 | 164 | 0 | NA | NA | NA |
| TCGA-02-0004-01 | TCGA-02-0004 | 59 | -21617 | 345 | 0 | 10,32 | Recurred/Progressed | NA |
| TCGA-08-0353-01 | TCGA-08-0353 | 58 | -21332 | 256 | 0 | 5,39 | Recurred/Progressed | NA |
| TCGA-12-1602-01 | TCGA-12-1602 | 58 | -21492 | 206 | 0 | 4,6 | Recurred/Progressed | NA |
| TCGA-02-0068-01 | TCGA-02-0068 | 57 | -21160 | 804 | 0 | 5,75 | Recurred/Progressed | NA |
| TCGA-41-6646-01 | TCGA-41-6646 | 73 | -26667 | NA | 0 | 6,57 | Recurred/Progressed | 4 |
| TCGA-28-1749-01 | TCGA-28-1749 | 73 | -26765 | NA | 0 | 9,2 | Recurred/Progressed | 1 |
| TCGA-19-1788-01 | TCGA-19-1788 | 39 | -14301 | 112 | 0 | NA | NA | NA |
| TCGA-02-0054-01 | TCGA-02-0054 | 44 | -16224 | 199 | 0 | 2,37 | Recurred/Progressed | NA |
| TCGA-32-2638-01 | TCGA-32-2638 | 67 | -24658 | NA | 0 | NA | NA | 0 |
| TCGA-06-0127-01 | TCGA-06-0127 | 67 | -24502 | 121 | 0 | 2,99 | Recurred/Progressed | NA |

Part 2

| CASE_ID | ETHNICITY | GENDER | HISTORY_NEOADJUVANT_TRTYN | ICD_10 | ICD_O_3_HISTOLOGY | ICD_O_3_SITE | INITIAL_PATHOLOGIC_DX_YEAR |
| --- | --- | --- | --- | --- | --- | --- | --- |
| TCGA-32-5222-01 | NOT HISPANIC OR LATINO | MALE | No | C71.9 | 9440/3 | C71.9 | 2010 |
| TCGA-27-1831-01 | NOT HISPANIC OR LATINO | MALE | No | C71.9 | 9440/3 | C71.9 | 2006 |
| TCGA-08-0531-01 | NOT HISPANIC OR LATINO | MALE | No | C71.9 | 9440/3 | C71.9 | 2005 |
| TCGA-06-0133-01 | NOT HISPANIC OR LATINO | MALE | No | C71.9 | 9440/3 | C71.9 | 2007 |
| TCGA-28-2506-01 | NOT HISPANIC OR LATINO | FEMALE | No | C71.9 | 9440/3 | C71.9 | 2009 |
| TCGA-28-1751-01 | NOT HISPANIC OR LATINO | FEMALE | No | C71.9 | 9440/3 | C71.9 | 2009 |
| TCGA-06-0125-01 | NOT HISPANIC OR LATINO | FEMALE | No | C71.9 | 9440/3 | C71.9 | 2001 |
| TCGA-12-3648-01 | NOT HISPANIC OR LATINO | FEMALE | No | C71.9 | 9440/3 | C71.9 | 2007 |
| TCGA-06-6389-01 | NOT HISPANIC OR LATINO | FEMALE | No | C71.9 | 9440/3 | C71.9 | 2011 |
| TCGA-12-1599-01 | NOT HISPANIC OR LATINO | FEMALE | No | C71.9 | 9440/3 | C71.9 | 2006 |
| TCGA-08-0349-01 | NOT HISPANIC OR LATINO | MALE | No | C71.9 | 9440/3 | C71.9 | 2000 |
| TCGA-12-0772-01 | NOT HISPANIC OR LATINO | MALE | No | C71.9 | 9440/3 | C71.9 | 2003 |
| TCGA-12-3650-01 | NOT HISPANIC OR LATINO | MALE | No | C71.9 | 9440/3 | C71.9 | 2008 |
| TCGA-12-0778-01 | NOT HISPANIC OR LATINO | MALE | No | C71.9 | 9440/3 | C71.9 | 2006 |
| TCGA-08-0525-01 | NOT HISPANIC OR LATINO | MALE | Yes | C71.9 | 9440/3 | C71.9 | 2001 |
| TCGA-02-0106-01 | NOT HISPANIC OR LATINO | MALE | No | C71.9 | 9440/3 | C71.9 | 2004 |
| TCGA-02-0115-01 | NOT HISPANIC OR LATINO | MALE | No | C71.9 | 9440/3 | C71.9 | 2006 |
| TCGA-12-0776-01 | NOT HISPANIC OR LATINO | MALE | No | C71.9 | 9440/3 | C71.9 | 2005 |
| TCGA-27-2518-01 | NOT HISPANIC OR LATINO | MALE | No | C71.9 | 9440/3 | C71.9 | 2007 |
| TCGA-12-0769-01 | NOT HISPANIC OR LATINO | MALE | No | C71.9 | 9440/3 | C71.9 | 2002 |
| TCGA-02-2485-01 | NOT HISPANIC OR LATINO | MALE | No | C71.9 | 9440/3 | C71.9 | 2009 |
| TCGA-08-0510-01 | NOT HISPANIC OR LATINO | MALE | No | C71.9 | 9440/3 | C71.9 | 2000 |
| TCGA-06-1087-01 | NOT HISPANIC OR LATINO | MALE | No | C71.9 | 9440/3 | C71.9 | 2008 |
| TCGA-41-2575-01 | NOT HISPANIC OR LATINO | MALE | No | C71.9 | 9440/3 | C71.9 | 2009 |
| TCGA-27-2519-01 | NOT HISPANIC OR LATINO | MALE | No | C71.9 | 9440/3 | C71.9 | 2009 |
| TCGA-02-0064-01 | NOT HISPANIC OR LATINO | MALE | No | C71.9 | 9440/3 | C71.9 | 2005 |
| TCGA-12-0618-01 | NOT HISPANIC OR LATINO | MALE | No | C71.9 | 9440/3 | C71.9 | 2003 |
| TCGA-08-0358-01 | NOT HISPANIC OR LATINO | MALE | No | C71.9 | 9440/3 | C71.9 | 2005 |
| TCGA-08-0244-01 | NOT HISPANIC OR LATINO | MALE | No | C71.9 | 9440/3 | C71.9 | 2001 |
| TCGA-32-2491-01 | NOT HISPANIC OR LATINO | MALE | No | C71.9 | 9440/3 | C71.9 | 2005 |
| TCGA-02-0075-01 | NOT HISPANIC OR LATINO | MALE | No | C71.9 | 9440/3 | C71.9 | 2005 |
| TCGA-27-2528-01 | NOT HISPANIC OR LATINO | MALE | No | C71.9 | 9440/3 | C71.9 | 2008 |
| TCGA-08-0348-01 | NOT HISPANIC OR LATINO | MALE | No | C71.9 | 9440/3 | C71.9 | 2000 |
| TCGA-02-0027-01 | NOT HISPANIC OR LATINO | FEMALE | No | C71.9 | 9440/3 | C71.9 | 2005 |
| TCGA-26-1442-01 | NOT HISPANIC OR LATINO | MALE | No | C71.9 | 9440/3 | C71.9 | 2008 |
| TCGA-08-0390-01 | NOT HISPANIC OR LATINO | MALE | No | C71.9 | 9440/3 | C71.9 | 2005 |
| TCGA-02-0070-01 | NOT HISPANIC OR LATINO | MALE | No | C71.9 | 9440/3 | C71.9 | 2006 |
| TCGA-12-0775-01 | NOT HISPANIC OR LATINO | FEMALE | No | C71.9 | 9440/3 | C71.9 | 2004 |
| TCGA-19-5951-01 | NOT HISPANIC OR LATINO | FEMALE | No | C71.9 | 9440/3 | C71.9 | 2010 |
| TCGA-12-3646-01 | NOT HISPANIC OR LATINO | FEMALE | No | C71.9 | 9440/3 | C71.9 | 2005 |
| TCGA-12-5295-01 | NOT HISPANIC OR LATINO | FEMALE | No | C71.9 | 9440/3 | C71.9 | 2008 |
| TCGA-02-0086-01 | NOT HISPANIC OR LATINO | FEMALE | No | C71.9 | 9440/3 | C71.9 | 2005 |
| TCGA-06-5858-01 | NOT HISPANIC OR LATINO | FEMALE | No | C71.9 | 9440/3 | C71.9 | 2010 |
| TCGA-06-0644-01 | NOT HISPANIC OR LATINO | MALE | No | C71.9 | 9440/3 | C71.9 | 2007 |
| TCGA-06-5414-01 | NOT HISPANIC OR LATINO | MALE | No | C71.9 | 9440/3 | C71.9 | 2010 |
| TCGA-06-6701-01 | NOT HISPANIC OR LATINO | MALE | No | C71.9 | 9440/3 | C71.9 | 2011 |
| TCGA-12-0620-01 | NOT HISPANIC OR LATINO | MALE | No | C71.9 | 9440/3 | C71.9 | 2005 |
| TCGA-06-6390-01 | NOT HISPANIC OR LATINO | MALE | No | C71.9 | 9440/3 | C71.9 | 2011 |
| TCGA-02-0004-01 | NOT HISPANIC OR LATINO | MALE | No | C71.9 | 9440/3 | C71.9 | 2002 |
| TCGA-08-0353-01 | NOT HISPANIC OR LATINO | MALE | No | C71.9 | 9440/3 | C71.9 | 2003 |
| TCGA-12-1602-01 | NOT HISPANIC OR LATINO | MALE | No | C71.9 | 9440/3 | C71.9 | 2008 |
| TCGA-02-0068-01 | NOT HISPANIC OR LATINO | MALE | No | C71.9 | 9440/3 | C71.9 | 2006 |
| TCGA-41-6646-01 | NOT HISPANIC OR LATINO | FEMALE | No | C71.9 | 9440/3 | C71.9 | 2011 |
| TCGA-28-1749-01 | NOT HISPANIC OR LATINO | MALE | No | C71.9 | 9440/3 | C71.9 | 2009 |
| TCGA-19-1788-01 | NOT HISPANIC OR LATINO | MALE | No | C71.9 | 9440/3 | C71.9 | 2009 |
| TCGA-02-0054-01 | NOT HISPANIC OR LATINO | FEMALE | No | C71.9 | 9440/3 | C71.9 | 2005 |
| TCGA-32-2638-01 | NOT HISPANIC OR LATINO | MALE | No | C71.9 | 9440/3 | C71.9 | 2009 |
| TCGA-06-0127-01 | NOT HISPANIC OR LATINO | MALE | No | C71.9 | 9440/3 | C71.9 | 2002 |

Part 3

| CASE_ID | KARNOFSKY_PERFORMANCE_SCORE | LAST_CONTACT_DAYS_TO | METHOD_OF_SAMPLE_PROCUREMENT | OS_MONTHS | OS_STATUS | PERFORMANCE_STATUS_TIMING |
| --- | --- | --- | --- | --- | --- | --- |
| TCGA-32-5222-01 | NA | 165 | Tumor resection | 19,22 | DECEASED | NA |
| TCGA-27-1831-01 | 80 | 505 | Tumor resection | 16,59 | DECEASED | Pre-Operative |
| TCGA-08-0531-01 | 80 | 168 | Tumor resection | 7,56 | DECEASED | Pre-Adjuvant Therapy |
| TCGA-06-0133-01 | NA | 428 | Tumor resection | 14,29 | DECEASED | NA |
| TCGA-28-2506-01 | 80 | 205 | Tumor resection | NA | DECEASED | Post-Adjuvant Therapy |
| TCGA-28-1751-01 | 80 | 232 | Tumor resection | NA | DECEASED | Post-Adjuvant Therapy |
| TCGA-06-0125-01 | 60 | 1439 | Tumor resection | 47,57 | DECEASED | Pre-Operative |
| TCGA-12-3648-01 | 80 | 784 | Excisional Biopsy | 26,91 | DECEASED | Post-Adjuvant Therapy |
| TCGA-06-6389-01 | 100 | 237 | Tumor resection | 7,79 | LIVING | Pre-Adjuvant Therapy |
| TCGA-12-1599-01 | 80 | 769 | Tumor resection | 25,66 | DECEASED | Pre-Adjuvant Therapy |
| TCGA-08-0349-01 | NA | 231 | Tumor resection | 9,79 | DECEASED | NA |
| TCGA-12-0772-01 | 80 | 1615 | Excisional Biopsy | 53,81 | DECEASED | Pre-Adjuvant Therapy |
| TCGA-12-3650-01 | 80 | 333 | Excisional Biopsy | 10,94 | DECEASED | Pre-Adjuvant Therapy |
| TCGA-12-0778-01 | 80 | 452 | Excisional Biopsy | 14,91 | DECEASED | Post-Adjuvant Therapy |
| TCGA-08-0525-01 | 80 | 486 | Tumor resection | 15,97 | DECEASED | Other |
| TCGA-02-0106-01 | 80 | 355 | Tumor resection | 11,66 | DECEASED | NA |
| TCGA-02-0115-01 | 80 | 476 | Tumor resection | 15,64 | DECEASED | NA |
| TCGA-12-0776-01 | 80 | 277 | Excisional Biopsy | 9,72 | DECEASED | Pre-Adjuvant Therapy |
| TCGA-27-2518-01 | 80 | 753 | Tumor resection | 24,74 | DECEASED | Pre-Operative |
| TCGA-12-0769-01 | 100 | 378 | Excisional Biopsy | 15,05 | DECEASED | Pre-Adjuvant Therapy |
| TCGA-02-2485-01 | 80 | 470 | Excisional Biopsy | NA | DECEASED | NA |
| TCGA-08-0510-01 | 80 | 107 | Tumor resection | 4,27 | DECEASED | Other |
| TCGA-06-1087-01 | 60 | 109 | Tumor resection | 4,04 | DECEASED | Pre-Operative |
| TCGA-41-2575-01 | 60 | 290 | Excisional Biopsy | 9,53 | DECEASED | Post-Adjuvant Therapy |
| TCGA-27-2519-01 | 80 | 316 | Tumor resection | NA | DECEASED | Pre-Operative |
| TCGA-02-0064-01 | 100 | 600 | Tumor resection | 19,71 | DECEASED | NA |
| TCGA-12-0618-01 | 60 | 50 | Excisional Biopsy | 12,98 | DECEASED | Pre-Adjuvant Therapy |
| TCGA-08-0358-01 | 80 | 594 | Tumor resection | 22,27 | DECEASED | Pre-Adjuvant Therapy |
| TCGA-08-0244-01 | 80 | 690 | Tumor resection | 22,67 | DECEASED | Other |
| TCGA-32-2491-01 | NA | 372 | Tumor resection | 12,22 | DECEASED | NA |
| TCGA-02-0075-01 | 80 | 634 | Tumor resection | 20,83 | DECEASED | NA |
| TCGA-27-2528-01 | 80 | 480 | Tumor resection | 15,77 | DECEASED | Pre-Operative |
| TCGA-08-0348-01 | 80 | 316 | Tumor resection | 12,16 | DECEASED | Other |
| TCGA-02-0027-01 | 100 | 315 | Tumor resection | 12,16 | DECEASED | NA |
| TCGA-26-1442-01 | 80 | 953 | Tumor resection | 31,31 | LIVING | Post-Adjuvant Therapy |
| TCGA-08-0390-01 | 60 | 322 | Tumor resection | 13,96 | DECEASED | Post-Adjuvant Therapy |
| TCGA-02-0070-01 | 80 | 762 | Tumor resection | NA | DECEASED | NA |
| TCGA-12-0775-01 | 60 | 167 | Excisional Biopsy | 7,62 | DECEASED | Post-Adjuvant Therapy |
| TCGA-19-5951-01 | 80 | 244 | Tumor resection | 8,02 | DECEASED | Pre-Adjuvant Therapy |
| TCGA-12-3646-01 | 80 | 1315 | Excisional Biopsy | 43,99 | DECEASED | Pre-Adjuvant Therapy |
| TCGA-12-5295-01 | NA | 399 | Tumor resection | 14,91 | DECEASED | Pre-Adjuvant Therapy |
| TCGA-02-0086-01 | 100 | 268 | Tumor resection | 8,8 | DECEASED | NA |
| TCGA-06-5858-01 | 100 | 187 | Tumor resection | NA | DECEASED | Pre-Adjuvant Therapy |
| TCGA-06-0644-01 | 80 | 375 | Tumor resection | 12,61 | DECEASED | Pre-Operative |
| TCGA-06-5414-01 | 80 | 273 | Tumor resection | NA | DECEASED | Pre-Operative |
| TCGA-06-6701-01 | NA | 151 | Tumor resection | 4,96 | LIVING | NA |
| TCGA-12-0620-01 | 100 | 181 | Excisional Biopsy | 10,45 | DECEASED | Pre-Adjuvant Therapy |
| TCGA-06-6390-01 | 80 | 164 | Tumor resection | 5,39 | DECEASED | Pre-Operative |
| TCGA-02-0004-01 | 80 | 345 | Tumor resection | 11,33 | DECEASED | NA |
| TCGA-08-0353-01 | 80 | 195 | Tumor resection | 8,41 | DECEASED | Post-Adjuvant Therapy |
| TCGA-12-1602-01 | 60 | 195 | Tumor resection | 6,77 | DECEASED | Pre-Adjuvant Therapy |
| TCGA-02-0068-01 | 80 | 804 | Tumor resection | 26,41 | DECEASED | NA |
| TCGA-41-6646-01 | NA | 236 | Tumor resection | 12,45 | DECEASED | Other |
| TCGA-28-1749-01 | 80 | 280 | Tumor resection | NA | DECEASED | Post-Adjuvant Therapy |
| TCGA-19-1788-01 | 60 | 112 | Tumor resection | 3,68 | DECEASED | Pre-Adjuvant Therapy |
| TCGA-02-0054-01 | 80 | 199 | Tumor resection | 6,54 | DECEASED | NA |
| TCGA-32-2638-01 | NA | 224 | Tumor resection | 25,16 | DECEASED | Pre-Adjuvant Therapy |
| TCGA-06-0127-01 | 60 | 109 | Tumor resection | 3,98 | DECEASED | Pre-Operative |

Part 4

| CASE_ID | RACE | SPECIMEN_SECOND_LONGEST_DIMENSION | TISSUE_SOURCE_SITE | TUMOR_STATUS | TUMOR_TISSUE_SITE | VITAL_STATUS |
| --- | --- | --- | --- | --- | --- | --- |
| TCGA-32-5222-01 | WHITE | 0,9 | 32 | WITH TUMOR | Brain | Alive |
| TCGA-27-1831-01 | WHITE | 1,5 | 27 | WITH TUMOR | Brain | Dead |
| TCGA-08-0531-01 | WHITE | 0,7 | 8 | WITH TUMOR | Brain | Dead |
| TCGA-06-0133-01 | WHITE | 0,9 | 6 | WITH TUMOR | Brain | Dead |
| TCGA-28-2506-01 | WHITE | 0,5 | 28 | WITH TUMOR | Brain | Alive |
| TCGA-28-1751-01 | WHITE | 0,9 | 28 | WITH TUMOR | Brain | Alive |
| TCGA-06-0125-01 | WHITE | 0,9 | 6 | WITH TUMOR | Brain | Dead |
| TCGA-12-3648-01 | WHITE | 0,7 | 12 | WITH TUMOR | Brain | Dead |
| TCGA-06-6389-01 | WHITE | 0,6 | 6 | WITH TUMOR | Brain | Alive |
| TCGA-12-1599-01 | WHITE | 0,6 | 12 | WITH TUMOR | Brain | Dead |
| TCGA-08-0349-01 |  | 0,7 | 8 | WITH TUMOR | Brain | Dead |
| TCGA-12-0772-01 | WHITE | 1 | 12 | WITH TUMOR | Brain | Dead |
| TCGA-12-3650-01 | WHITE | 0,9 | 12 | WITH TUMOR | Brain | Dead |
| TCGA-12-0778-01 | WHITE | 0,8 | 12 | WITH TUMOR | Brain | Dead |
| TCGA-08-0525-01 |  | 0,7 | 8 | WITH TUMOR | Brain | Dead |
| TCGA-02-0106-01 | WHITE | 0,6 | 2 | WITH TUMOR | Brain | Dead |
| TCGA-02-0115-01 | ASIAN | 0,6 | 2 | WITH TUMOR | Brain | Dead |
| TCGA-12-0776-01 | WHITE | 0,5 | 12 | WITH TUMOR | Brain | Dead |
| TCGA-27-2518-01 | WHITE | 1 | 27 | WITH TUMOR | Brain | Dead |
| TCGA-12-0769-01 | WHITE | 0,7 | 12 | WITH TUMOR | Brain | Dead |
| TCGA-02-2485-01 | BLACK OR AFRICAN AMERICAN | 2 | 2 | WITH TUMOR | Brain | Alive |
| TCGA-08-0510-01 | WHITE | 1 | 8 | WITH TUMOR | Brain | Dead |
| TCGA-06-1087-01 | WHITE | 0,6 | 6 | WITH TUMOR | Brain | Dead |
| TCGA-41-2575-01 | WHITE | 0,9 | 41 | WITH TUMOR | Brain | Dead |
| TCGA-27-2519-01 | WHITE | 0,4 | 27 | WITH TUMOR | Brain | Alive |
| TCGA-02-0064-01 | WHITE | 0,8 | 2 | WITH TUMOR | Brain | Dead |
| TCGA-12-0618-01 | WHITE | 0,6 | 12 | NA | Brain | Dead |
| TCGA-08-0358-01 | WHITE | 0,8 | 8 | WITH TUMOR | Brain | Dead |
| TCGA-08-0244-01 | WHITE | 0,8 | 8 | WITH TUMOR | Brain | Dead |
| TCGA-32-2491-01 | WHITE | 0,3 | 32 | WITH TUMOR | Brain | Dead |
| TCGA-02-0075-01 | WHITE | 0,7 | 2 | WITH TUMOR | Brain | Dead |
| TCGA-27-2528-01 | WHITE | 0,7 | 27 | WITH TUMOR | Brain | Dead |
| TCGA-08-0348-01 | WHITE | 0,6 | 8 | WITH TUMOR | Brain | Dead |
| TCGA-02-0027-01 | WHITE | NA | 2 | WITH TUMOR | Brain | Dead |
| TCGA-26-1442-01 | WHITE | 1,3 | 26 | WITH TUMOR | Brain | Alive |
| TCGA-08-0390-01 | WHITE | 0,8 | 8 | WITH TUMOR | Brain | Dead |
| TCGA-02-0070-01 | WHITE | 0,6 | 2 | WITH TUMOR | Brain | Alive |
| TCGA-12-0775-01 | BLACK OR AFRICAN AMERICAN | 0,5 | 12 | WITH TUMOR | Brain | Dead |
| TCGA-19-5951-01 | WHITE | 0,7 | 19 | WITH TUMOR | Brain | Dead |
| TCGA-12-3646-01 | WHITE | 0,7 | 12 | WITH TUMOR | Brain | Dead |
| TCGA-12-5295-01 | WHITE | 0,9 | 12 | WITH TUMOR | Brain | Dead |
| TCGA-02-0086-01 | WHITE | 1 | 2 | WITH TUMOR | Brain | Dead |
| TCGA-06-5858-01 | WHITE | 0,9 | 6 | WITH TUMOR | Brain | Alive |
| TCGA-06-0644-01 | BLACK OR AFRICAN AMERICAN | 0,6 | 6 | WITH TUMOR | Brain | Alive |
| TCGA-06-5414-01 | WHITE | 1,1 | 6 | WITH TUMOR | Brain | Alive |
| TCGA-06-6701-01 | WHITE | 1 | 6 | WITH TUMOR | Brain | Alive |
| TCGA-12-0620-01 | WHITE | 0,2 | 12 | WITH TUMOR | Brain | Dead |
| TCGA-06-6390-01 | WHITE | 1 | 6 | WITH TUMOR | Brain | Dead |
| TCGA-02-0004-01 | WHITE | 1 | 2 | WITH TUMOR | Brain | Dead |
| TCGA-08-0353-01 | WHITE | 0,8 | 8 | WITH TUMOR | Brain | Dead |
| TCGA-12-1602-01 | WHITE | 0,6 | 12 | WITH TUMOR | Brain | Dead |
| TCGA-02-0068-01 | WHITE | 1,1 | 2 | WITH TUMOR | Brain | Dead |
| TCGA-41-6646-01 | WHITE | 0,9 | 41 | WITH TUMOR | Brain | Alive |
| TCGA-28-1749-01 | WHITE | 0,4 | 28 | WITH TUMOR | Brain | Alive |
| TCGA-19-1788-01 | WHITE | 0,7 | 19 | NA | Brain | Dead |
| TCGA-02-0054-01 | WHITE | NA | 2 | WITH TUMOR | Brain | Dead |
| TCGA-32-2638-01 | WHITE | 0,6 | 32 | TUMOR FREE | Brain | Alive |
| TCGA-06-0127-01 | WHITE | 0,9 | 6 | WITH TUMOR | Brain | Dead |
