# Supplementary material for: A 4-miRNA signature predicts the therapeutic outcome of glioblastoma
Source: Oncotarget. 2016 Jun 11;7(29):45764–75. doi: 10.18632/oncotarget.9945 (PMC5216759; doi:10.18632/oncotarget.9945)
Supplement: Supplementary file 3 [file oncotarget-07-45764-s003.docx]

| **reactomeID** | **numGenes** | **avgLFC** | **sdLFC** | **zValue** | **strength** | **pvalue** | **reactomeName** | **padjust** | **reactomeResult.genes.in.pathway** |
| --- | --- | --- | --- | --- | --- | --- | --- | --- | --- |
| 382551 | 15 | 0,400227732 | 0,027736221 | 14,42978574 | 1,550075342 | 7,39604E-18 | **Homo sapiens: Transmembrane transport of small molecules** | 1,81539E-17 | ABCB1, ADCY9, ADD2, AQP9, FTL, GABRB3, HK2, SLC1A2, SLC35C1, SLC38A3, SLC38A5, SLC39A4, SLC8A2, TCIRG1, TRPV2 |
| 168249 | 15 | 0,369591478 | 0,030182598 | 12,24518451 | 1,431421638 | 7,28089E-17 | **Homo sapiens: Innate Immune System** | 1,6382E-16 | ADCY9, CFD, CTSB, DUSP3, EREG, ICAM3, ITPR1, LCP2, MYO10, NFATC1, PPP3CB, PROS1, PSMB10, XRCC6 |
| 1474244 | 15 | 0,377464255 | 0,032595006 | 11,58043215 | 1,461912772 | 1,58296E-16 | **Homo sapiens: Extracellular matrix organization** | 3,05286E-16 | ADAM8, COL4A2, COL4A6, CTSB, ICAM3, ITGA3, ITGB3, LAMB3, MATN4, MMP7, PLOD2, PLOD3, SDC2, SPP1, THBS1 |
| 422475 | 12 | 0,383391412 | 0,033502915 | 11,44352408 | 1,328106809 | 3,19162E-13 | **Homo sapiens: Axon guidance** | 5,06905E-13 | COL4A2, EVL, ITGB3, ITSN1, MYL9, MYO10, PRNP, SCN5A, SDC2, SEMA6D, SOS2, SPTBN2 |
| 166520 | 11 | 0,388213427 | 0,034297451 | 11,31901676 | 1,287558275 | 4,28524E-12 | **Homo sapiens: Signalling by NGF** | 6,42786E-12 | ADCY9, ARHGEF6, BRAF, CRKL, DUSP3, EREG, HDAC2, ITPR1, ITSN1, PCSK5, SOS2 |
| 1266738 | 13 | 0,380294727 | 0,033964262 | 11,19690819 | 1,371172139 | 3,44994E-14 | **Homo sapiens: Developmental Biology** | 5,82177E-14 | CDON, COL4A2, EVL, ITGB3, ITSN1, MYL9, MYO10, PRNP, SCN5A, SDC2, SEMA6D, SOS2, SPTBN2 |
| 112316 | 11 | 0,390402173 | 0,037216871 | 10,48992467 | 1,294817525 | 9,12085E-12 | **Homo sapiens: Neuronal System** | 1,23132E-11 | ADCY9, BRAF, CAMK2D, COMT, GABRB3, GNG5, HCN3, KCNG4, KCNK3, MAOA, SLC1A2 |
| 388396 | 20 | 0,39061513 | 0,040156656 | 9,72728244 | 1,746883969 | 1,71448E-20 | **Homo sapiens: GPCR downstream signaling** | 5,14343E-20 | ADCY9, ADM, ADRA2B, ARHGEF6, EDN3, F2RL1, GAL, HEBP1, HRH1, HTR2B, ITPR1, ITSN1, OR2B2, OR2V2, OR4A5, OR4N4, OR51D1, PTGIR, SOS2 |
| 372790 | 21 | 0,388238921 | 0,040626382 | 9,5563253 | 1,779134245 | 2,42816E-21 | **Homo sapiens: Signaling by GPCR** | 9,36577E-21 | ADCY9, ADM, ADRA2B, ARHGEF6, EDN3, F2RL1, GAL, HEBP1, HRH1, HTR2B, ITPR1, ITSN1, OR2B2, OR2V2, OR4A5, OR4N4, OR51D1, PPP3CB, PTGIR, SOS2 |
| 195721 | 15 | 0,377876355 | 0,040096092 | 9,424269046 | 1,463508831 | 2,76772E-15 | **Homo sapiens: Signaling by Wnt** | 4,9819E-15 | AKT2, CCDC88C, CHD8, CUL3, HIST1H4L, HIST3H3, ITPR1, NFATC1, PPP3CB, PSMB10, SNX3, SOX4, TLE1, TNKS2 |
| 168256 | 35 | 0,383372727 | 0,040993906 | 9,35194438 | 2,268063637 | 6,74589E-35 | **Homo sapiens: Immune System** | 6,0713E-34 | ADCY9, AMICA1, AP1S3, CAMK2D, CD40, CFD, CRKL, CTSB, DUSP3, EREG, FCGR2B, HLA-DOB, ICAM3, IL1R1, IL1RN, IL3, ISG20, ITPR1, KIF26A, LCP2, LILRB2, LILRB3, MAP3K8, MID1, MYO10, NFATC1, PDCD1, PPP3CB, PROS1, PSMB10, SEC61A2, SOCS3, SPTBN2, XRCC6 |
| 194315 | 11 | 0,385139078 | 0,041541278 | 9,271238036 | 1,277361813 | 3,10325E-11 | **Homo sapiens: Signaling by Rho GTPases** | 3,98989E-11 | ARHGEF6, B9D2, EVL, H3F3A, HIST1H3A, HIST1H4L, IQGAP1, ITSN1, MYL9, RHOH, SOS2 |
| 1280218 | 17 | 0,385837041 | 0,044565886 | 8,657676806 | 1,590846875 | 1,10433E-16 | **Homo sapiens: Adaptive Immune System** | 2,29361E-16 | AMICA1, AP1S3, CD40, EREG, FCGR2B, HLA-DOB, ICAM3, ITPR1, KIF26A, LCP2, LILRB2, LILRB3, MAP3K8, PDCD1, PSMB10, SEC61A2, SPTBN2 |
| 74160 | 25 | 0,391504107 | 0,045397095 | 8,623990297 | 1,957520535 | 2,97371E-24 | **Homo sapiens: Gene Expression** | 2,00725E-23 | CCNT1, CPSF3, EIF3D, EIF3G, EIF3J, H3F3A, HDAC2, HIST1H3A, HIST1H4L, HNRNPA1, HSPB1, NPPA, NR1I3, NR3C1, PARP1, PLRG1, POLRMT, PSMB10, RPL22, RPL3, RPL4, SF3A3, SP1, TBP |
| 2262752 | 11 | 0,393870133 | 0,045885856 | 8,583693724 | 1,306319447 | 6,65248E-11 | **Homo sapiens: Cellular responses to stress** | 8,16441E-11 | CABIN1, CAMK2D, CBX6, EHMT2, H3F3A, HIST1H3A, HIST1H4L, HIST3H3, RAD50, SOD3, SP1 |
| 556833 | 21 | 0,404725123 | 0,047177868 | 8,578707365 | 1,854683513 | 2,05288E-20 | **Homo sapiens: Metabolism of lipids and lipoproteins** | 5,54278E-20 | ABHD5, ACADS, ACOX2, ALOX5AP, CBR1, CYP11A1, CYP7B1, ETNK2, FADS1, FDFT1, FDPS, GLA, GPD1L, LBR, PI4KA, PITPNB, PLD4, SP1, SPHK2, SPTLC3, SUMF1 |
| 162582 | 66 | 0,390170286 | 0,045621467 | 8,552339731 | 3,169758389 | 1,01586E-62 | **Homo sapiens: Signal Transduction** | 2,74282E-61 | ADCY9, ADM, ADRA2B, AKT2, ARHGEF6, B9D2, BRAF, CCDC88C, CCNT1, CDON, CHD8, COL4A2, CRKL, CUL3, DUSP3, EDN3, EREG, EVC, EVL, F2RL1, FAS, FRS3, GAL, H3F3A, HDAC2, HEBP1, HIST1H3A, HIST1H4L, HIST3H3, HRH1, HSPB1, HTR2B, IQGAP1, ITGB3, ITPR1, ITSN1, MYL9, NFATC1, OR2B2, OR2V2, OR4A5, OR4N4, OR51D1, PARP1, PCSK5, PDK3, PPP3CB, PSMB10, PTGIR, RHOH, SDC2, SNX3, SOCS3, SOS2, SOX4, SP1, SPP1, TCIRG1, TGFBR2, THBS1, TLE1, TNFRSF1A, TNKS2, VEGFB |
| 201681 | 11 | 0,382860413 | 0,044863723 | 8,533852976 | 1,269804337 | 7,04677E-11 | **Homo sapiens: TCF dependent signaling in response to WNT** | 8,27229E-11 | AKT2, CCDC88C, CHD8, CUL3, HIST1H4L, HIST3H3, PSMB10, SOX4, TLE1, TNKS2 |
| 109582 | 22 | 0,393077638 | 0,047714353 | 8,238142544 | 1,843697548 | 5,37265E-21 | **Homo sapiens: Hemostasis** | 1,81327E-20 | ADRA2B, AKAP1, AMICA1, CEACAM8, CFD, H3F3A, HDAC2, HIST1H3A, ITGA3, ITGB3, ITPR1, KIF26A, LCP2, PROCR, PROS1, PTGIR, SIRPA, SLC8A2, STXBP3, THBS1, VEGFB |
| 1643685 | 23 | 0,382109932 | 0,047346357 | 8,070524463 | 1,832534856 | 9,8822E-22 | **Homo sapiens: Disease** | 4,44699E-21 | AP1S3, CAMP, CCNT1, CYP11A1, EIF2AK2, EPM2A, EREG, H3F3A, HIST1H3A, HIST1H4L, NPPA, PSMB10, RPL22, RPL3, RPL4, SDC2, TBP, TCIRG1, TGFBR2, TNKS2, XRCC6 |
| 500792 | 10 | 0,398109734 | 0,051234282 | 7,770377909 | 1,258933517 | 1,46758E-09 | **Homo sapiens: GPCR ligand binding** | 1,58499E-09 | ADM, ADRA2B, EDN3, F2RL1, GAL, HEBP1, HRH1, HTR2B, PTGIR |
| 71387 | 10 | 0,389212264 | 0,050589733 | 7,693503005 | 1,230797247 | 1,60302E-09 | **Homo sapiens: Metabolism of carbohydrates** | 1,66468E-09 | CHST6, CHST7, EPM2A, HK2, PFKM, PYGL, SDC2, SGSH, UGP2 |
| 1430728 | 55 | 0,399195528 | 0,052765816 | 7,565419374 | 2,960513269 | 1,46456E-49 | **Homo sapiens: Metabolism** | 1,97716E-48 | ABCB1, ABHD5, ACADS, ACOX2, ADCY9, ALOX5AP, AOX1, CA12, CA2, CBR1, CHST6, CHST7, COMT, CYP11A1, CYP2A6, CYP2E1, CYP7B1, EPM2A, ETFA, ETNK2, FADS1, FAH, FDFT1, FDPS, GCDH, GLA, GNG5, GPD1L, GPHN, HK2, IQGAP1, ITPKB, ITPR1, LBR, MAOA, NAT1, NT5E, PDK3, PFKM, PI4KA, PITPNB, PLD4, PSMB10, PYGL, SDC2, SDHD, SGSH, SP1, SPHK2, SPTLC3, SUMF1, UGP2, UPP1, UQCRH |
| 5663205 | 13 | 0,389875456 | 0,054788216 | 7,11604588 | 1,405715948 | 7,48447E-12 | **Homo sapiens: Infectious disease** | 1,06358E-11 | AP1S3, CAMP, CCNT1, EIF2AK2, PSMB10, RPL22, RPL3, RPL4, TBP, TCIRG1, XRCC6 |
| 392499 | 26 | 0,402100754 | 0,059906674 | 6,712119442 | 2,050319589 | 1,59889E-22 | **Homo sapiens: Metabolism of proteins** | 8,63403E-22 | DPH3, EIF3D, EIF3G, EIF3J, ENPEP, GGCX, HDGF, IGFBP6, MAN1A1, MGAT4A, MGAT4B, PARP1, PIGT, PROS1, RPL22, RPL3, RPL4, SAMM50, SEC61A2, SEMA6D, STAG2, SUMF1, THBS1, TOMM70A |
| 1640170 | 10 | 0,394626902 | 0,063315207 | 6,232734906 | 1,247919835 | 1,03292E-08 | **Homo sapiens: Cell Cycle** | 1,03292E-08 | B9D2, H3F3A, HIST1H3A, HIST1H4L, HIST3H3, HUS1, PSMB10, RAD50, STAG2, USO1 |
| 597592 | 12 | 0,409946535 | 0,072211664 | 5,67701274 | 1,420096455 | 6,40302E-10 | **Homo sapiens: Post-translational protein modification** | 7,2034E-10 | DPH3, GGCX, MAN1A1, MGAT4A, MGAT4B, PARP1, PIGT, PROS1, SEMA6D, STAG2, SUMF1, THBS1 |
