# Supplementary material for: A 4-miRNA signature predicts the therapeutic outcome of glioblastoma
Source: Oncotarget. 2016 Jun 11;7(29):45764–75. doi: 10.18632/oncotarget.9945 (PMC5216759; doi:10.18632/oncotarget.9945)
Supplement: Supplementary file 4 [file oncotarget-07-45764-s004.docx]

| **HGNC_symbol** | **correlation_coefficient** |
| --- | --- |
| CHD8 | 0,387572511 |
| TOB2 | 0,42906137 |
| RMND5B | -0,344005512 |
| ETFA | -0,451437906 |
| POLRMT | 0,354933441 |
| NENF | -0,355804175 |
| SDK2 | 0,362912578 |
| SOCS4 | 0,388609983 |
| ZC3H14 | 0,339185487 |
| LOC442229 | -0,34265218 |
| DDX21 | 0,376676035 |
| LYPD2 | -0,378116546 |
| UQCRH | -0,377387851 |
| VEGFB | -0,345555035 |
| ARVCF | 0,405138523 |
| FBXO16 | -0,410549957 |
| CEACAM8 | -0,351729135 |
| PI4KA | 0,34859543 |
| LASS6 | 0,393598698 |
| CDH17 | -0,445778019 |
| CYP2A6 | -0,481851483 |
| C19orf42 | -0,371956156 |
| CLDN12 | -0,397019081 |
| OR4A5 | 0,372488901 |
| HTR2B | -0,373115338 |
| TOX4 | 0,373675129 |
| ZNF766 | 0,388162596 |
| PATZ1 | 0,457863026 |
| ANKRD29 | -0,370697849 |
| DSCR3 | -0,449389733 |
| UGP2 | -0,34472598 |
| HEMK1 | -0,403752066 |
| HDGF | 0,370714752 |
| C21orf121 | -0,345063751 |
| CRKL | 0,362625397 |
| EDG5 | -0,390178851 |
| EIF3G | 0,404068538 |
| PITPNB | 0,413004407 |
| PRR3 | 0,376465907 |
| TFIP11 | 0,427416006 |
| GZMM | -0,343118232 |
| OBP2A | -0,449078464 |
| LOC124512 | -0,364708384 |
| ZNF549 | 0,366592479 |
| DHX16 | 0,342607216 |
| KIAA0586 | 0,356853708 |
| CBX6 | 0,352844055 |
| NR1I3 | -0,421183417 |
| PHF21B | 0,34523606 |
| SPACA1 | -0,403009575 |
| KCNG4 | -0,472228329 |
| PROCA1 | -0,378558028 |
| TULP2 | -0,364338331 |
| HDHD1A | -0,40634883 |
| TMEM136 | -0,475138642 |
| CBR3 | -0,407588279 |
| HELB | 0,381551182 |
| XPNPEP3 | 0,411008733 |
| MYCN | 0,344461543 |
| KLF13 | 0,392243489 |
| PHB | -0,381280469 |
| COMT | 0,385642623 |
| TUG1 | 0,351650747 |
| GPR101 | -0,391181468 |
| SMARCB1 | 0,356473218 |
| IL17RE | -0,339774792 |
| YY1 | 0,402534708 |
| FAM80A | -0,343958498 |
| C19orf29 | 0,42189688 |
| TMEM55B | 0,384152794 |
| C4orf40 | 0,371162171 |
| ADRA2B | -0,414986614 |
| LOC90835 | -0,353829225 |
| DKFZP434B0335 | 0,42540021 |
| MICAL3 | 0,374870052 |
| LOC554235 | -0,364920867 |
| CCDC88C | 0,392745857 |
| LOC441376 | -0,407815557 |
| FAM134B | 0,342457578 |
| F2RL1 | 0,374909011 |
| SLMO1 | -0,34860089 |
| SAMM50 | 0,395638242 |
| MRPL15 | -0,519276936 |
| OR2B2 | -0,427382408 |
| EIF3D | 0,395124338 |
| SDHD | -0,352278527 |
| ZNF136 | 0,338557654 |
| OR11H12 | 0,35407561 |
| C1orf201 | -0,380118682 |
| TMEM162 | -0,46341485 |
| RPL3 | 0,33891644 |
| EIF4ENIF1 | 0,365380971 |
| SPATA18 | -0,567968232 |
| CABIN1 | 0,444947884 |
| DGCR14 | 0,403935582 |
| CXorf40B | -0,345647419 |
| LOC388965 | -0,339096278 |
| LOC643406 | -0,35098014 |
| TEKT5 | -0,339285696 |
| C20orf39 | 0,346525178 |
| GLRX2 | -0,350302373 |
| XRCC6 | 0,359125159 |
| LOH12CR1 | -0,352632453 |
| MPO | -0,344137003 |
| ZHX3 | 0,340005439 |
| SALL1 | 0,389230703 |
| SLC10A7 | 0,357524703 |
| MAOA | 0,357427292 |
| LOC158381 | 0,383344352 |
| ZNF350 | 0,369306389 |
| ZCCHC12 | -0,434040708 |
| CASC4 | 0,421341306 |
| TMSL8 | -0,44509392 |
| NDUFAF1 | 0,467315551 |
| LOC147650 | 0,449586808 |
| FAM84B | 0,340719804 |
| C19orf18 | 0,394041932 |
| AKT2 | 0,345544164 |
| EIF3J | 0,481301041 |
| LOC253970 | -0,342021404 |
| TOX2 | -0,378108516 |
| RPP25 | -0,481847154 |
| FAM128A | -0,363358107 |
| KIAA0143 | 0,398194641 |
| NDP | 0,484669146 |
| TMEM147 | 0,449766018 |
| ZFP106 | 0,380475634 |
| LRRC16 | 0,45997353 |
| FAM82C | 0,451550687 |
| THSD1 | 0,344906261 |
| ABCB1 | 0,398693657 |
| CPSF3 | -0,422117394 |
| SLC26A4 | 0,440515249 |
| PFKM | 0,34381227 |
| PLEKHF2 | 0,398019671 |
| ATG9B | -0,387304006 |
| CLDN12 | 0,353479579 |
| KIF26A | -0,339502648 |
| ZNF226 | 0,407065673 |
| C9orf125 | -0,342211419 |
| GLB1L3 | -0,369545076 |
| TSPAN6 | 0,349166091 |
| MATN2 | 0,356956847 |
| ARHGEF6 | 0,375696967 |
| ANKIB1 | 0,375359072 |
| DYX1C1 | 0,36990968 |
| SHC4 | 0,339732035 |
| STXBP3 | 0,457067381 |
| SLC38A5 | 0,402448988 |
| FAM63B | 0,360975639 |
| RP13-36C9.6 | 0,488804931 |
| CTSL2 | -0,363886225 |
| FUBP1 | -0,462138685 |
| LAMB3 | 0,359680989 |
| TEX10 | -0,395818756 |
| C20orf54 | 0,340128484 |
| ZFAND6 | 0,409029815 |
| B9D2 | 0,340187176 |
| GCDH | 0,3559684 |
| FOXD2 | -0,366572349 |
| DAZAP2 | 0,39630685 |
| SIRPA | 0,355650804 |
| GPRIN1 | -0,413428006 |
| SLC38A3 | 0,408052491 |
| SEMA6D | 0,406178494 |
| FBXL7 | 0,34095518 |
| DHX58 | 0,342974625 |
| PDLIM5 | 0,372423364 |
| SPHK2 | 0,451835888 |
| HYAL3 | -0,384236819 |
| TMEM45A | -0,429285826 |
| CAMK2D | 0,370146935 |
| CCHCR1 | -0,344796775 |
| C10orf65 | 0,425607687 |
| NR3C1 | 0,413540627 |
| EDN3 | 0,343084724 |
| CYP7B1 | -0,375544639 |
| OSBPL6 | 0,366193275 |
| TLE1 | 0,447579848 |
| PLD4 | 0,389365861 |
| HK2 | -0,416501206 |
| CA2 | 0,361502571 |
| KIAA1913 | -0,356296634 |
| SNX3 | 0,392303245 |
| WDR67 | -0,353844869 |
| NPTN | 0,359627519 |
| NANOS1 | -0,358495371 |
| MARVELD2 | 0,444199891 |
| SCN5A | -0,372101972 |
| EPB41L4B | -0,382492402 |
| KCNK3 | -0,348552524 |
| ETNK2 | -0,339704382 |
| DSCR6 | -0,351964799 |
| GAS2L1 | 0,346602715 |
| CDC42EP3 | -0,370152975 |
| C2orf34 | 0,352235204 |
| TLK1 | 0,349478753 |
| SIX2 | -0,37997785 |
| GPD1L | 0,39225838 |
| MATN4 | -0,374735008 |
| ITPKB | 0,376479264 |
| PBXIP1 | 0,379857878 |
| ZHX1 | 0,360109891 |
| ZNF404 | 0,377602081 |
| PASK | -0,344463864 |
| LOC81691 | -0,370481385 |
| C6orf203 | 0,414375484 |
| MYO10 | 0,400022513 |
| MID1 | 0,449223107 |
| PPTC7 | 0,345844513 |
| CDADC1 | 0,403913893 |
| ZFHX4 | 0,367840688 |
| SLC1A2 | 0,383487055 |
| ZNF259 | -0,347103379 |
| USP53 | 0,358181449 |
| PRNP | 0,38558962 |
| PRPF3 | 0,398784486 |
| PLRG1 | -0,368019201 |
| CCDC76 | 0,343540195 |
| DDX26B | 0,343549346 |
| GPKOW | -0,392647214 |
| ELP3 | -0,359266216 |
| U1SNRNPBP | -0,383411881 |
| SFRS11 | 0,431042099 |
| DMTF1 | 0,392557329 |
| OR1D5 | -0,415274392 |
| CCNT1 | 0,348714154 |
| LOC389517 | 0,3815012 |
| VPS13A | 0,342248338 |
| UBE2G2 | 0,402908686 |
| VAMP3 | 0,372668034 |
| LOC284402 | -0,433900272 |
| ACTR8 | -0,354115877 |
| C4orf29 | 0,361851349 |
| SF3A3 | 0,360771308 |
| tcag7.1017 | 0,352070143 |
| EIF2AK2 | 0,408763236 |
| HLA-DOB | 0,497811054 |
| LRRC27 | -0,375444373 |
| ZNF2 | -0,344081809 |
| DKFZp434K191 | 0,486008124 |
| CSTF2T | -0,390341677 |
| CHD8 | 0,404814886 |
| DDB1 | 0,408022024 |
| MMP7 | -0,398449905 |
| KIAA1486 | 0,360685994 |
| OR2V2 | -0,381742345 |
| EVC | -0,354995813 |
| KIAA1199 | -0,34300607 |
| POU3F1 | 0,351948481 |
| STK17A | -0,376956482 |
| EIF1 | -0,355007456 |
| CHST6 | -0,506065977 |
| SDC2 | -0,342288448 |
| EIF3EIP | 0,36841584 |
| RPL4 | 0,347450095 |
| EMR1 | -0,428097701 |
| HUS1 | -0,373297191 |
| ITSN1 | 0,363477955 |
| TNKS2 | 0,344924264 |
| CADPS2 | -0,395924176 |
| HIST1H4L | 0,354672948 |
| ZDHHC22 | 0,511805206 |
| KIAA1546 | 0,365419603 |
| BTBD14A | -0,530512581 |
| PROS1 | -0,349885059 |
| SOCS4 | 0,420292519 |
| SOCS3 | -0,368045476 |
| GAL | -0,340201845 |
| PIGT | -0,422809059 |
| STH | 0,350706191 |
| MMP24 | -0,442936502 |
| C14orf24 | 0,440902063 |
| RBM4B | 0,483434739 |
| C1orf96 | 0,3403509 |
| MAN1A1 | -0,375263702 |
| ABHD5 | -0,346496407 |
| ALOX5AP | -0,398206919 |
| SLC8A2 | 0,38644945 |
| EPC2 | 0,37886295 |
| EFHD2 | -0,351536672 |
| tcag7.23 | 0,339947044 |
| CCNB1IP1 | 0,443306368 |
| ENPEP | -0,342030862 |
| C8orf31 | 0,437540936 |
| RAD50 | -0,347352937 |
| HRH1 | -0,430309827 |
| PRKRIP1 | -0,432294272 |
| SPTBN2 | 0,422629249 |
| C14orf173 | -0,380286494 |
| COL4A6 | -0,348376505 |
| TOMM70A | 0,442535364 |
| NUDT16P | -0,342857062 |
| BRMS1L | 0,353613214 |
| ICAM3 | -0,357130887 |
| ADAM8 | -0,406143655 |
| PTGFRN | -0,345250031 |
| NONO | 0,345577803 |
| ECEL1P2 | -0,375212408 |
| FNDC3B | -0,399200099 |
| FER1L3 | -0,386344476 |
| SEC61A2 | 0,428228248 |
| RABL5 | -0,375081493 |
| CASP4 | -0,384787926 |
| PDK3 | -0,368124223 |
| BRD3 | 0,368770342 |
| AOX1 | -0,355954893 |
| IL1R1 | -0,360894571 |
| TBC1D8B | -0,359151528 |
| XPO7 | 0,352618104 |
| FLJ23834 | 0,363000342 |
| LBR | 0,356848026 |
| ERGIC1 | -0,445550102 |
| GPHN | 0,546094276 |
| FLJ21062 | -0,364471277 |
| GAL3ST3 | 0,388545109 |
| ARVCF | 0,384922239 |
| FAM20A | -0,388911527 |
| MSN | -0,414535667 |
| EPDR1 | -0,377193922 |
| ATAD2B | 0,400058522 |
| STAG2 | 0,53815433 |
| ZFP37 | 0,366326883 |
| PELO | -0,511754086 |
| REP15 | -0,349632586 |
| TIPARP | -0,37116784 |
| SECISBP2 | 0,340049658 |
| HDDC3 | -0,369844239 |
| CD40 | -0,347623123 |
| EHMT2 | 0,455095873 |
| MAG1 | -0,362873483 |
| KRTAP17-1 | 0,437096052 |
| HDGFRP3 | 0,350207269 |
| FLJ22662 | -0,413534531 |
| MAP3K8 | -0,362128354 |
| STC1 | -0,338990737 |
| C9orf95 | -0,365495139 |
| TTC5 | 0,349415212 |
| TCTN1 | -0,356440914 |
| PPP2R5C | 0,525830106 |
| CAPG | -0,539808196 |
| SLC26A4 | -0,363321396 |
| EREG | -0,360655048 |
| MCAM | -0,347171589 |
| CAMP | -0,467368966 |
| PTX3 | -0,437461521 |
| GLYCTK | -0,413323234 |
| AYTL2 | -0,4195752 |
| MR1 | -0,484131802 |
| ZBTB6 | 0,426394819 |
| PPP1R3A | 0,371153235 |
| PRRT3 | -0,423462403 |
| C1QL4 | 0,347837414 |
| TMEM67 | -0,36287214 |
| DIRAS3 | -0,385746675 |
| RBM12 | 0,413091136 |
| H2AFJ | -0,360314442 |
| KIF26A | 0,347548128 |
| B3GALT4 | -0,360782602 |
| GADD45G | 0,380938452 |
| MSL-1 | 0,423458275 |
| CDYL2 | -0,396019825 |
| HTR2B | -0,358159849 |
| PYGL | -0,406736758 |
| AKAP1 | 0,476676005 |
| PSORS1C1 | -0,345696217 |
| SP1 | -0,435353413 |
| SERPINB1 | -0,404727537 |
| IQGAP1 | -0,406448406 |
| SIL1 | -0,43790695 |
| PATZ1 | 0,584465984 |
| ISG20 | -0,45476142 |
| ANKRD29 | -0,394926113 |
| EPM2A | 0,36547972 |
| FAH | -0,418067794 |
| MAP2 | 0,356561078 |
| SLC25A19 | -0,376361372 |
| ZNF34 | 0,443161863 |
| DSCR3 | -0,350671323 |
| PI4KAP2 | 0,388180815 |
| MKRN3 | 0,506677905 |
| CBLN3 | -0,386294297 |
| FLJ13236 | -0,348675111 |
| DENND1C | -0,391898506 |
| MSL2L1 | 0,423955246 |
| TGFBR2 | -0,345470152 |
| KIAA1727 | 0,371591296 |
| UGP2 | -0,406812753 |
| PCSK5 | -0,374806944 |
| LOC200810 | -0,354418934 |
| KIF21B | 0,402632659 |
| CRIP3 | 0,349984252 |
| ACOX2 | -0,41041248 |
| MFSD9 | -0,403002082 |
| NXPH1 | 0,342399758 |
| ANP32B | 0,408610071 |
| RAB43 | -0,414997636 |
| ADCY9 | -0,413476445 |
| MTMR11 | -0,369620519 |
| ADD2 | 0,418927147 |
| RP11-679B17.1 | 0,400104505 |
| PRR3 | 0,383683978 |
| CBR1 | -0,451324261 |
| C6orf162 | 0,343271995 |
| WASF1 | 0,455589727 |
| HSPB1 | -0,405188023 |
| ACY1L2 | 0,34967092 |
| SH3BP4 | 0,3956163 |
| ANKRD13B | 0,572278364 |
| ADCK1 | 0,385997112 |
| SUSD2 | -0,462687314 |
| FADS1 | 0,425363904 |
| MGAT4B | -0,346916449 |
| LRRC8D | 0,39118761 |
| RAB35 | -0,344083593 |
| ZNF24 | 0,373879669 |
| PHF16 | 0,379094884 |
| LIF | -0,369199343 |
| SUV39H2 | 0,431162318 |
| ENDOG | 0,383651433 |
| ZNF549 | 0,363804657 |
| NAG18 | 0,377821023 |
| GLA | -0,391094165 |
| STK40 | -0,471689458 |
| SCYE1 | -0,34373179 |
| SEPT3 | 0,488253131 |
| FAM22D | 0,356423418 |
| CSTA | -0,364888064 |
| NPPA | 0,356075548 |
| RALGPS1 | 0,460792037 |
| PHF21B | 0,494018596 |
| PAFAH1B3 | 0,411895529 |
| FLJ22222 | -0,360210488 |
| BCKDK | -0,381541344 |
| CTSB | -0,382108054 |
| CMYA5 | -0,438855487 |
| TXNDC3 | -0,356980081 |
| FAM3B | -0,45592124 |
| C7orf49 | -0,342298434 |
| SPP1 | -0,449178049 |
| FDFT1 | 0,442224007 |
| ARFGEF2 | -0,415286084 |
| IL21R | -0,402613966 |
| FBXL10 | 0,41639737 |
| ACOT9 | -0,398880216 |
| FRS3 | 0,396539963 |
| RUFY1 | -0,384621606 |
| PTGIR | -0,480515679 |
| PLOD2 | -0,341267105 |
| PI3 | -0,461407918 |
| LOC55565 | 0,384526495 |
| ACCN4 | 0,348884132 |
| TULP2 | -0,403574976 |
| LYPLA3 | -0,390966706 |
| C2orf18 | -0,392760369 |
| COL4A2 | -0,372908148 |
| HEBP1 | -0,477350208 |
| H3F3A | 0,407455156 |
| DYRK3 | -0,448309702 |
| PUM2 | 0,411846957 |
| PPP3CB | 0,340714738 |
| LILRB3 | -0,420049182 |
| CXorf6 | 0,364981629 |
| BTN2A2 | -0,417686282 |
| IGFBP6 | -0,368477762 |
| SLC37A2 | -0,501104179 |
| ITFG3 | -0,395067861 |
| C10orf137 | 0,341259187 |
| VENTX | -0,355666035 |
| C14orf65 | 0,44439054 |
| CHST7 | -0,357079738 |
| MYCN | 0,553016937 |
| GGCX | -0,477596838 |
| KIAA1543 | 0,339811327 |
| IL1RN | -0,404337344 |
| CYB5R2 | -0,405186769 |
| GABRB3 | 0,345969602 |
| HOXD3 | 0,383869311 |
| LCP2 | -0,371230107 |
| SEZ6L | 0,386696527 |
| PDCD1 | -0,367376243 |
| OR51D1 | -0,352402063 |
| ADM | -0,388464243 |
| KIAA1128 | 0,408833312 |
| USO1 | -0,424265604 |
| RPL10L | 0,358145645 |
| KCNIP2 | 0,434779039 |
| ELK3 | -0,473492114 |
| NAT1 | -0,34255624 |
| LEPREL1 | -0,475527092 |
| HNRNPA1 | 0,344457548 |
| EVL | 0,458188536 |
| CFD | -0,339444064 |
| C20orf116 | -0,348924873 |
| AMICA1 | -0,364586981 |
| FAM129A | -0,359739691 |
| UBQLN4 | 0,404674635 |
| SMARCB1 | 0,3832385 |
| GNG5 | -0,422149016 |
| RCAN2 | -0,436596836 |
| DNALI1 | -0,406460594 |
| OR4N4 | 0,4130092 |
| RPL22 | 0,488143069 |
| NOX4 | -0,351382574 |
| CYP2E1 | 0,349432521 |
| TM7SF4 | -0,431235521 |
| C20orf29 | -0,414188367 |
| LCTL | -0,424681374 |
| PMFBP1 | -0,396881547 |
| TBP | 0,338754423 |
| IL3 | 0,348691401 |
| DRAM | -0,436881852 |
| ACADS | -0,442434611 |
| MS4A6E | -0,350307236 |
| BATF | -0,446781631 |
| C11orf30 | 0,354437501 |
| PARP1 | 0,364350928 |
| FAM60A | 0,367241581 |
| SHD | 0,464925253 |
| HNRPCL1 | 0,350841232 |
| MLLT6 | 0,391689888 |
| LOC90835 | -0,48591117 |
| YIPF1 | -0,375898164 |
| TNFAIP8 | -0,37852826 |
| UBXD7 | 0,34622167 |
| BRSK2 | 0,48775749 |
| C17orf28 | 0,345847912 |
| HDAC2 | 0,452048473 |
| KCNK15 | -0,396479586 |
| TCIRG1 | -0,439978488 |
| GRN | -0,381815943 |
| TRIP4 | -0,454909931 |
| MGAT4A | -0,344823023 |
| HFE | -0,340884359 |
| ACTG2 | -0,383490039 |
| UPP1 | -0,558520485 |
| MICALL2 | -0,363128748 |
| ITGB3 | -0,344742466 |
| PSCDBP | -0,462384143 |
| HIST1H3A | 0,455312486 |
| PSMB10 | -0,351717156 |
| UNC45A | -0,340889633 |
| FDPS | 0,346658556 |
| SNX21 | -0,554738742 |
| FLRT1 | 0,462167597 |
| SPOCD1 | -0,4058523 |
| CYP11A1 | -0,387813731 |
| NAGPA | -0,494700568 |
| LOC441376 | -0,429759052 |
| ZFP161 | 0,355532628 |
| AQP9 | -0,382205822 |
| SRPK1 | 0,428135501 |
| NFIA | 0,37841437 |
| PCDH21 | 0,442112374 |
| LHFPL2 | -0,383632627 |
| PTPN22 | -0,440176828 |
| ITGA3 | -0,41254121 |
| WDR89 | 0,434693413 |
| KIAA1609 | -0,341362152 |
| HIST3H3 | 0,353854034 |
| ABHD9 | -0,351860397 |
| SAMM50 | 0,354698907 |
| HSF2 | 0,519740348 |
| RHOH | -0,342520682 |
| TNFAIP6 | -0,377826443 |
| FAM26B | -0,39687222 |
| FLJ33790 | 0,384307954 |
| CATSPER1 | -0,463089867 |
| HPS1 | -0,456888745 |
| TNFRSF1A | -0,530526676 |
| C9orf140 | 0,399756483 |
| SUMF1 | -0,536901206 |
| BCL7A | 0,451900237 |
| NT5E | -0,348972458 |
| ZNF136 | 0,351833471 |
| MGP | -0,371932705 |
| CUL3 | 0,350941007 |
| C1orf201 | -0,357661436 |
| ZNF18 | -0,372718278 |
| SPTLC3 | -0,417786913 |
| C6orf170 | 0,359140521 |
| KLHDC2 | 0,405375503 |
| FSD1 | 0,442707645 |
| CA12 | -0,421171429 |
| TRIM6 | -0,375200992 |
| FLJ36874 | 0,34123304 |
| SPEG | -0,428286604 |
| FAM3C | -0,358326567 |
| AP1S3 | -0,455560928 |
| SLC39A4 | -0,430841936 |
| C12orf49 | -0,411681843 |
| KIAA0408 | 0,376113171 |
| DUSP3 | -0,448744829 |
| FAHD2B | -0,356248876 |
| CHD7 | 0,361621526 |
| FTHP1 | -0,406193408 |
| CDON | 0,343134511 |
| RAB2B | 0,416785401 |
| SPINK5L3 | -0,413562599 |
| C14orf104 | 0,349441798 |
| RPL3 | 0,377933512 |
| THBS1 | -0,416548159 |
| SPATA18 | -0,425923086 |
| DKFZP586H2123 | -0,370397989 |
| SGSH | -0,40261979 |
| SLC35C1 | -0,348061311 |
| MYL9 | -0,364089442 |
| CXorf40B | -0,426183909 |
| SPIN3 | 0,405545259 |
| CSTB | -0,36150648 |
| TLOC1 | 0,341970487 |
| PCOLCE2 | -0,347281446 |
| BRAF | -0,387286646 |
| MGC14376 | -0,446944353 |
| PROCR | -0,415184111 |
| RFXDC2 | 0,40486165 |
| FOLR3 | -0,344776153 |
| TTC26 | -0,420461349 |
| ZNF22 | 0,429988964 |
| BCL2L12 | -0,349225355 |
| CXCL14 | -0,406639037 |
| TMEM111 | -0,490502559 |
| RAB42 | -0,358972326 |
| SOX4 | 0,477097869 |
| C8orf58 | -0,383701569 |
| FCGR2B | -0,402403477 |
| DKFZp434B1231 | 0,420811182 |
| NFATC1 | -0,360613914 |
| ZBTB22 | 0,40811146 |
| DPH3 | -0,339931173 |
| FAS | -0,501828485 |
| SOD3 | -0,355535743 |
| XRCC6 | 0,345955305 |
| SOS2 | 0,3684801 |
| C7orf42 | -0,40748489 |
| TMCC2 | 0,359396537 |
| MARCH5 | 0,342443363 |
| FTL | -0,397640909 |
| TRPV2 | -0,430681477 |
| GATS | 0,414736453 |
| VIM | -0,39816964 |
| ITPR1 | -0,363048888 |
| SHPRH | 0,383106945 |
| GARNL3 | 0,352844647 |
| AOF2 | 0,404516764 |
| TMEM169 | 0,510418057 |
| TNS1 | -0,411595441 |
| ACPP | -0,457667386 |
| PLOD3 | -0,355865234 |
| MFSD1 | -0,387635857 |
| HCN3 | 0,408057438 |
| C6orf117 | -0,338883786 |
| C1orf113 | -0,442945803 |
| USP37 | 0,355767667 |
